# Self-correcting mismatches in metastable hydrogen-bonded organic frameworks with an 11-fold interpenetrated array†

**DOI:** 10.1039/d4sc02751e

**Published:** 2024-08-06

**Authors:** Guomin Xia, Chunlei Zhou, Xingliang Xiao, Yang Yang, Fuqing Yu, Hongming Wang

**Affiliations:** a College of Chemistry and Chemical Engineering, Nanchang University Nanchang 330031 China hongmingwang@ncu.edu.cn; b Jiangxi Provincial Key Laboratory of Functional Crystalline Materials Chemistry Nanchang 330031 China guominxia@ncu.edu.cn; c Institute for Advanced Study, Nanchang University Nanchang 330031 China

## Abstract

The polymorphic self-correction from a metastable phase to a stable one often occurs and plays crucial roles in synthesizing robust hydrogen-bonded organic frameworks (HOFs). However, identifying metastable phases and understanding the self-correcting mechanisms is a challenging venture due to their intrinsic instability. Here, we for the first time introduce 1,8-naphtholactam (Np) as a hydrogen-bonding synthon positioned on the periphery of a bicarbazole to create a versatile molecular unit for 3D HOFs. The as-synthesized NCU-HOF1, analyzed by single-crystal X-ray diffraction (SCXRD), is found to be metastable. It exhibits an 11-fold interpenetrated dia topology with a quarter of the Np units exhibiting monomeric N–H⋯O interactions between adjacent Np link sites, which readily self-correct upon desolvation to form fully dimeric ones. Consequently, the resultant NCU-HOF1a becomes highly robust in polar solvents, strong acid or alkaline aqueous solutions, and has permanent porosity with contracted cavities for selective adsorption and efficient “turn-up” fluorescent sensing of C_2_H_4_ gas. This work not only debuts a new hydrogen-bonding synthon but offers more insights into investigating solid-state dynamics in metastable HOFs.

## Abstract 2

SCXRD data reveal a nanoconfinement isomerization in 11-fold interpenetrated NCU-HOFs, resulting in highly robust structures with permanent porosity, contracted cavities for selective adsorption, and efficient fluorescent sensing of C_2_H_4_ gas.

## Graphical abstract

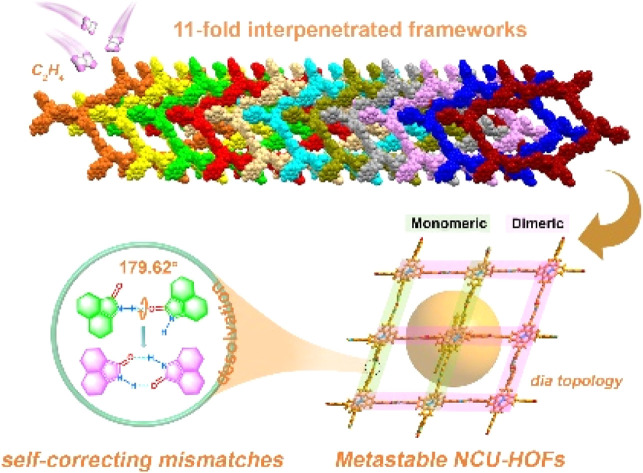

## Introduction

Hydrogen-bonded organic frameworks (HOFs) represent a rapidly evolving class of synthetic crystalline porous materials with substantial potential spanning diverse scientific disciplines.^1–7^ Their distinctive attributes, encompassing porosity, tunability, sustainability, and practicality, render them compelling candidates for a myriad of applications, including gas adsorption and separation,^8–10^ proton conduction,^11,12^ heterogeneous catalysis,^13,14^ biomedical applications,^15–17^ and beyond. Generally, HOFs are self-assembled by the interaction of organic molecule units through non-covalent hydrogen-bonds.^18,19^ In this regard, the carboxylic acid,^20–22^ diaminotriazine (DAT),^23,24^ pyrazole,^25,26^ imidazole,^27,28^ benzimidazolone,^29,30^ and charge-assisted ionic hydrogen bonding patterns^31–33^ have been employed in constructing HOF archetypes. In the past decade, careful selection of building blocks and functional group modifications within specific organic molecular units have led to significant advancements in the geometry of HOFs. These advancements encompass aspects such as shape, size, symmetry, and dimensions, as well as surface chemistry^1–5,34–40^ underscoring the crucial role of pre-design strategies.

However, the exploration of HOFs has presented a substantial challenge in implementing reticular chemistry for the self-assembly of hydrogen-bonding units. Hydrogen bonds exhibit significantly weaker (10–40 kJ mol^−1^), more flexible, and less directional bonding energies and angles compared to coordination (90–350 kJ mol^−1^) and covalent bonds (300–600 kJ mol^−1^).^1,41^ These intrinsic characteristics of hydrogen bonds in HOFs enable mild synthesis conditions,^42,43^ solution processability,^42,44^ and ease of regeneration,^45,46^ yet simultaneously result in the facile formation of metastable structures and complexity.^47–49^ The arrangement of hydrogen bonds among organic molecule units is susceptible to disruption by solvent disturbances, leading to the formation of (non)porous networks. These networks typically exhibit undesired hydrogen bonding between donor and acceptor groups, as well as fractured hydrogen bonds interacting with solvent molecules.^50–54^ This situation would be aggravated within highly interpenetrated HOFs, where the interpenetration of frameworks promotes synergistic interactions (π–π stacking, Ar/C–H⋯π interactions, van der Waals interactions)^18,55,56^ among organic molecule units to further stabilize these mismatched hydrogen bonds. It is thus believed that investigating the solid-state dynamics of mismatched hydrogen bond patterns within metastable HOFs would contribute to deeply deciphering their self-assembly and evolution, yet it remains an ongoing dilemma.

**Fig. 1:**
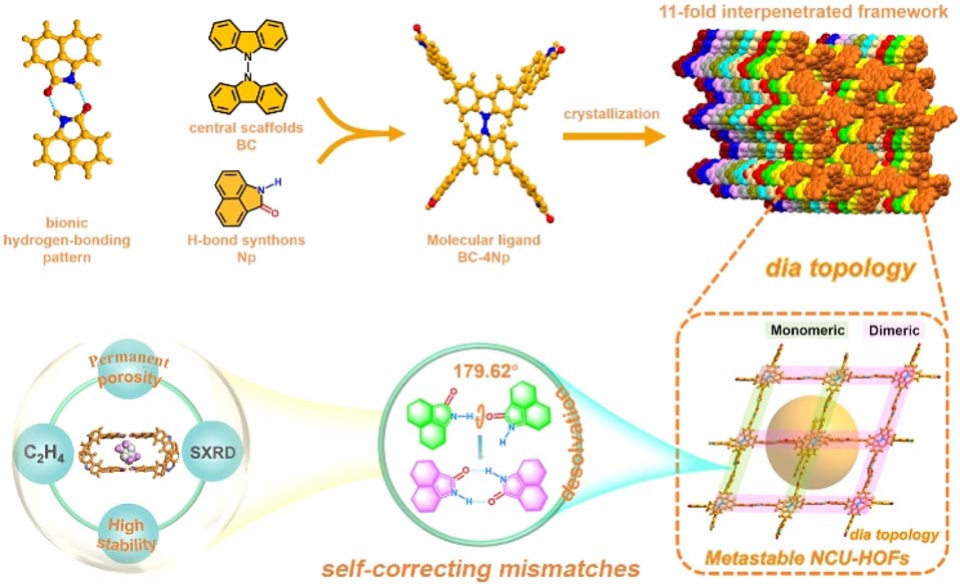
Schematic diagram illustrating the self-correcting mismatches within NCU-HOFs constructed from the BC–4Np molecular ligand.

In this study, we fabricated 3D HOFs by the self-assembly of organic molecule units consisting of peripheral 1,8-naphtholactam (Np) and central bicarbazole in solution. The selection of the Np synthon was based on its fluorescence behaviors and its capacity to form stable dimers through intermolecular hydrogen bonding, as recently reported in our laboratory.^57,58^ Utilizing single-crystal X-ray diffraction (SCXRD) data, we present, for the first time, the self-correcting mismatches of hydrogen bond patterns in an 11-fold interpenetrated array of metastable HOF1 upon desolvation (Fig. 1). This involved a remarkable “cross-door” rotation of nearly 180°between adjacent frameworks at the Np link sites with monomeric N–H⋯O interactions. The ensuing permanent porosity with contracted cavities, coupled with structural robustness and bright green emission of the activated NCU-HOFs were then illustrated, enabling the selective adsorption and efficient “turn-up” fluorescent sensing of ethylene(C_2_H_4_) gas.

## Results and discussion

**Fig. 2:**
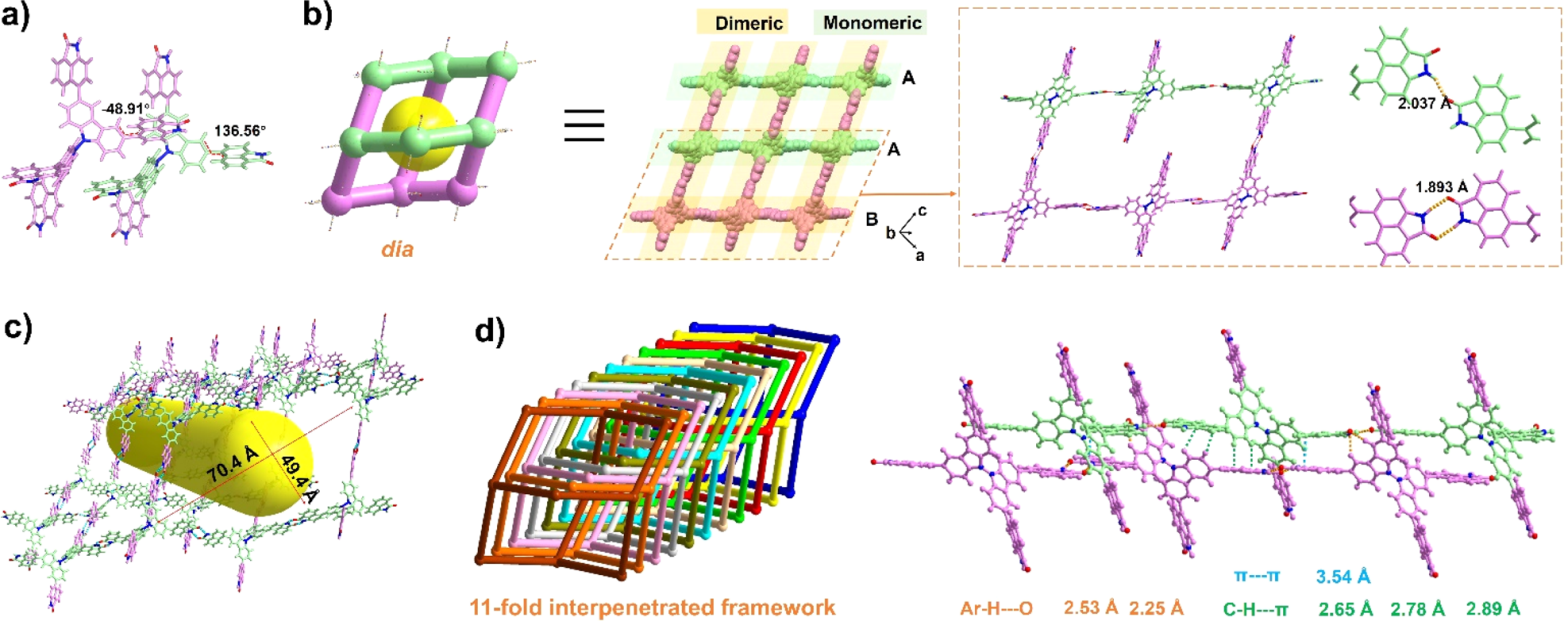
(a) Distinct molecular configurations of BC–4Np. (b) Alternating monomeric and dimeric N–H⋯O hydrogen bonds between adjacent BC–4Np molecules. (c) Void space in the dia topology in NCU-HOF1. (d) The 11-fold interpenetrated topological frameworks (different colors represent different frameworks) and side view of the stacking. The purple structure represents the dimer formed, and the mint green one represents the monomer.

The organic ligand BC–4Np was synthesized through a Suzuki coupling reaction involving 3,3′,6,6′-tetrabromo-9,9′-bicarbazole (BC–4Br) and boric acid esterified Np (Np-pin). Comprehensive characterization of its molecular structure was achieved using ^1^H NMR, ^13^C NMR, and mass spectroscopy, yielding satisfactory results (details in the ESI†). Block-shaped single crystals were obtained *via* vapor diffusion of acetone into a solution of BC–4Np in *N*,*N*′-dimethylformamide (DMF) at 298 K. SCXRD analysis disclosed that NCU-HOF1 crystallizes in the monoclinic space group *I*2/*a* (Table S1†). Within NCU-HOF1, two crystallographically unique BC–4Np molecules exist in the asymmetric unit (Fig. 2a). Distinct hydrogen bonding modes between adjacent Np linkers were observed within each BC–4Np unit, featuring partially monomeric and fully dimeric N–H⋯O hydrogen bonds in a 1 : 2 ratio, with distances ranging from 1.89 to 2.04 Å, forming a single dia 3D network (Fig. 2b). The substantial void space of 70.4 × 49.4 Å^2^ in this single dia 3D network facilitates the formation of a dia{6^6^} topological structure with an 11-fold interpenetrated array (Fig. 2c and d). This interpenetration is primarily facilitated by multiple Ar–H⋯O, Ar–H⋯π, and π–π interactions between Np and adjacent BC/Np moieties in BC–4Np units (Fig. S1 and S2†). These observations underscore the pivotal role of the large π-planar configuration of the Np synthon in the highly interpenetrated array of NCU-HOF1. Despite the 11-fold interpenetration, serrated 1D pore channels along the *c*-axis in NCU-HOF1 persist, with cages measuring 9.0 × 9.6 Å^2^ and necks as small as 5.3 Å (type I, Fig. S3†). Additionally, isolated cavities with cross-sections of approximately 7.6 × 7.0 Å^2^ were detected in NCU-HOF1 (type II, Fig. S3†). The total solvent-accessible void of NCU-HOF1 was estimated to be around 30% using PLATON.^59^

**Fig. 3:**
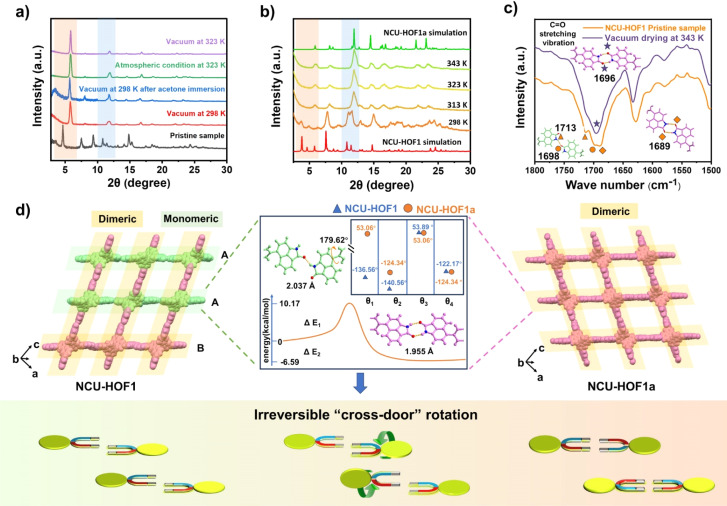
(a) PXRD variations of NCU-HOF1 upon desolvation (vacuum: −1 bar). (b) Variable-temperature PXRD patterns of degraded NCU-HOF1 under a N_2_ atmosphere. (c) *In situ* FTIR spectrum of NCU-HOF1. (d) Schematic diagram illustrating the self-correcting N–H⋯O interactions between adjacent Np link sites through an irreversible “cross-door” rotation pathway. Inset: The difference in dihedral angles between the carbazole and Np segments, and the energy profile from monomer to dimer formation of BC–4Np in NCU-HOF1.

During the activation process, a noteworthy phenomenon was observed in the desolvation of the as-synthesized sample (NCU-HOF1) under high vacuum to generate the activated sample (NCU-HOF1a). The powder X-ray diffraction (PXRD) patterns displayed significant changes, including a shift in the strongest diffraction peak of NCU-HOF1 from 3.1° to 5.2° and a reduction in the number of diffraction peaks, indicative of a structural transformation during desolvation (Fig. S4†). Subsequent tests revealed that elevated temperature and/or vacuum conditions induced a phase transition in NCU-HOF1 (Fig. 3a). Variable-temperature PXRD patterns demonstrated that the phase transition occurred at 313 K, despite being degenerate (Fig. 3b). This observation was corroborated by thermogravimetric analysis (TGA) and differential scanning calorimetry (DSC) data under nitrogen (N_2_) conditions (Fig. S5†), suggesting the facile escape of disordered solvent molecules from the frameworks. Fourier transform infrared spectroscopy (FTIR) further indicated the structural transformation of NCU-HOF1, as evidenced by the disappearance of the absorption peak at 1713 cm^−1^, establishing a close correlation with the hydrogen bond receptor C=O in Np synthons (Fig. 3c).

Through acetone immersion and vacuum outgassing at 298 K, the guest-free NCU-HOF1a crystal structure is successfully obtained for X-ray diffraction analysis, employing a single-crystal-to-single-crystal (SCSC) transformation approach. SCXRD analysis reveals that NCU-HOF1a crystallizes in the monoclinic space group *C*2/*c*, maintaining an 11-fold interpenetrated array with a slight lattice volume contraction in the same dia topology (Table S1 and Fig. S6 and S7†). In contrast to the metastable NCU-HOF1, all BC–4Np molecules in NCU-HOF1a exhibit higher structural symmetry and become crystallographically equivalent. This transformation is associated with the conversion of N–H⋯O interactions between adjacent Np link sites from being partially monomeric to completely dimeric upon activation (Fig. 3d). This observation aligns with the (FTIR) data, underscoring the significance of the hydrogen-bonding pattern in this self-correcting process. A detailed analysis reveals a “cross-door” rotation of two Np units in A–A layers, reaching an angle of nearly 180° (Fig. S8†). Simultaneously, two BC–4Np molecules at the adjacent B layer slide towards each other in opposite directions, maintaining a distance of approximately 4.6 Å. This sliding is primarily induced by the C–H⋯O interaction between the neighboring Np units at layers A and B, generating π-stacking in the frameworks. The self-correction dynamics, characterized by a dramatic rotation and slip of assembly units within the 11-fold interpenetrated HOFs, represent a remarkable and hitherto unreported phenomenon.

**Fig. 4:**
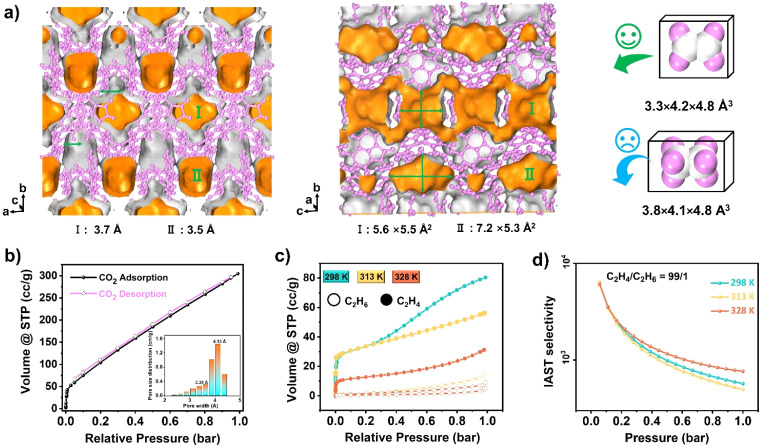
(a) Framework with two pore channels in NCU-HOF1a and the schematic diagram of the size-dependent separation for C_2_H_4_ and C_2_H_6_ molecules. (b) Gas adsorption and desorption curves for CO_2_ in NCU-HOF1a at 273 K. (c) Gas adsorption isotherms for C_2_H_4_ (hollow circles) and C_2_H_6_ (solid circles) in NCU-HOF1a at 298 K (cyan), 318 K (orange), and 333 K (dull red). (d) IAST selectivity of NCU-HOF1a for C_2_H_4_ (99 : 1, v/v) at 298 K (cyan), 318 K (orange), and 333 K (dull red).

In the context of energy considerations, the transformation of monomeric Np units into dimers occurs through rotation around the N–H⋯O bond, exhibiting an energy barrier of 10.17 kcal mol^−1^ and an exothermic process of 6.57 kcal mol^−1^ (Fig. 3d middle). When the free N–H of Np units is stabilized by guest acetone molecules, rotation becomes unlikely due to the presence of a higher energy barrier of 28.30 kcal mol^−1^ and an exothermic energy of 5.79 kcal mol^−1^ (Fig. S9†). The theoretical analysis concurs with the experimental observation that NCU-HOF1 can be easily activated into NCU-HOF1a through heating and/or vacuum conditions, as it removes guest acetone molecules. Following activation, the serrated 1D pore channel of the HOFs contracts into a dispersive 0D pore, resulting in a reduction of the void pore volume to approximately 20%. Furthermore, NCU-HOF1a crystals exhibit two types of irregular cavities (I and II), primarily surrounded by Np synthons (Fig. 4a). Along the *c*-axis, cavity I features the largest cages measuring 5.5 × 5.6 Å^2^ with the smallest necks of 3.7 Å, while cavity II comprises cages of 5.3 × 7.2 Å^2^ with the smallest necks of 3.5 Å (Fig. S10†). These dimensions align more favorably with the size of small C_2_H_4_ molecules (3.3 × 4.2 × 4.8 Å^3^) than ethane (C_2_H_6_) molecules (3.8 × 4.1 × 4.8 Å^3^),^8^ providing additional motivation for exploring potential capture and separation within these contracted cavities.

To assess chemical stability, NCU-HOF1a underwent treatment in common organic solvents (methanol, acetonitrile, acetone, trichloromethane, and toluene), HCl (pH = 1), and NaOH (pH = 13) solutions at 298 K. After one month, the framework retained its structural integrity, as confirmed by PXRD patterns, scanning electron microscope (SEM) images, and fluorescence photographs (Fig. S11 and S12†), revealing its high structural robustness. Variable-temperature PXRD patterns demonstrated NCU-HOF1a's exceptional thermal stability, with the decomposition temperature confirmed to be above 578 K by thermogravimetric analysis in an N_2_ atmosphere (Fig. S13†). It is noteworthy that the conversion of NCU-HOF1a to NCU-HOF1 could not be achieved, even when soaked in the mother solution for sample preparation, confirming its thermodynamic stability (Fig. S14†). This irreversibility aligns with crystallographic data both before and after the structural transformation, underscoring the self-correction of the mismatched hydrogen-bonding pattern. Meanwhile, NCU-HOF1a exhibited a maximum emission wavelength (*λ*_em_) at 526 nm with a fluorescence quantum yield (*Φ*_PL_) of up to 36.1% (Fig. S15†). The bright yellow fluorescence of NCU-HOF1a remained unchanged after immersion in common organic solvents and strong acid or alkali water solutions. The fluorescence decay profiles fit well with a double-exponential function, characterized by short lifetimes of approximately 4.79 ns (Fig. S15†). These fluorescence features, mainly inherited from Np synthons, can be sustained and are highly consistent with chemical stability. Notably, NCU-HOF1a represents the first instance of high-efficiency fluorescence derived from hydrogen-bonding synthons, with fluorescence efficiencies comparable to those reported in previous HOF literature (Table S2†).

The permanent porosity of NCU-HOF1a was established through carbon dioxide (CO_2_) gas sorption experiments at 273 K. In Fig. 4b, the adsorbed CO_2_ amount in the first step, at *P*/*P*_0_ = 0.001, is approximately 40 cm^3^ g^−1^, indicative of a micropore filling process. The total CO_2_ uptake at 1 bar reaches 300 cm^3^ g^−1^, corresponding to a total pore volume of 0.122 cm^3^ g^−1^, consistent with the theoretical value calculated from the crystal structure (0.103 cm^3^ g^−1^). A small hysteresis loop suggests that HOFs effectively preserve pore characteristics during the adsorption/desorption process. The Brunauer–Emmett–Teller (BET) surface area of NCU-HOF1a was calculated to be 538 m^2^ g^−1^ (Fig. S16†), comparable to reported counterparts with an 11-fold interpenetrated array.^60^ The pore size distribution, calculated using non-localized density functional theory (NL-DFT), exhibited main peaks at 3.38 and 4.13 Å, consistent with values obtained from the crystal structure using the Poreblazer program (Fig. 4b and S17†). N_2_ isotherms at 77 K revealed a characteristic reversible type-III isotherm for NCU-HOF1a (Fig. S18†), indicating an extremely weak interaction between NCU-HOF1a and N_2_ gas.

Single-component adsorption isotherms of C_2_H_6_ and C_2_H_4_ for NCU-HOF1a were examined under ambient conditions (Fig. 4c and S19 and Table S3†). The total C_2_H_4_ uptake at 298 K and 1 bar was 80.93 cm^3^ g^−1^ (3.61 mmol g^−1^), with a second adsorption step at 0.4 bar indicating dual adsorption sites for C_2_H_4_, one of which exhibits preferential adsorption. Conversely, a type-IV isotherm was detected for C_2_H_6_ adsorption, and the total C_2_H_6_ uptake was only 14.80 cm^3^ g^−1^ (0.66 mmol g^−1^) under identical conditions. Such a preferential adsorption of C_2_H_4_ over C_2_H_6_ is accompanied by a large adsorption ratio for C_2_H_4_/C_2_H_6_ (5.46), suggesting the potential of NCU-HOF1a for separating these two gases. Additionally, the total C_2_H_4_ uptake decreased with rising temperature, and the second adsorption step vanished at 313 K and 328 K. Calculations of the adsorption selectivity of NCU-HOF1a for C_2_H_4_/C_2_H_6_ (50/50, 90/10 and 99/1) mixtures using ideal adsorbed solution theory (IAST) revealed high C_2_H_4_ selectivity for mixtures at 298 K and 1 bar (Fig. 4d and S20 and Table S3†). The initial value of the experimental isosteric heat of adsorption (*Q*_st_) for C_2_H_4_ on NCU-HOF1a was calculated to be 20 kJ mol^−1^, indicating superior adsorption/desorption processes compared to traditional metals or π coordination gas adsorbing materials.^61^

**Fig. 5:**
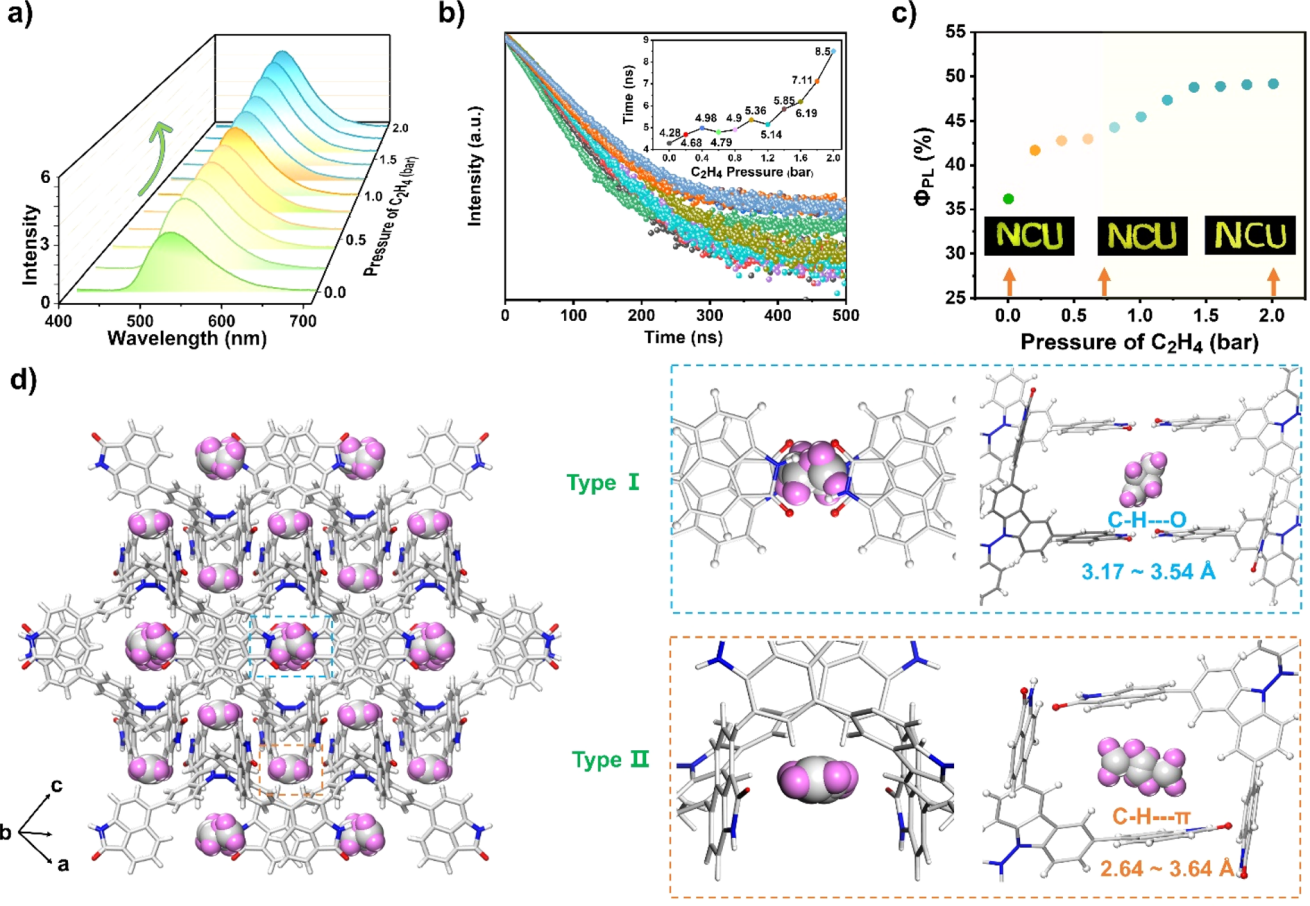
Changes in (a) fluorescence intensity, (b) lifetime profiles, and (c) *Φ*_PL_ of NCU-HOF1a upon C_2_H_4_ uptake (pressure ranging from 0 to 2 bar). (d) Multiple intermolecular interactions between C_2_H_4_ gas and Np synthons in the framework of NCU-HOF1a.

The fluorescence behavior of NCU-HOF1a before and after C_2_H_4_ uptake was then recorded. As shown in Fig. 5a–c, the as-prepared NCU-HOF1a@C_2_H_4_ (for preparation details see the ESI†) maintained bright yellow-green fluorescence at *λ*_em_ = 526 nm, with *Φ*_PL_ and lifetime gradually increasing from 36.1 to 49.1% and from 4.28 to 8.50 ns upon C_2_H_4_ uptake. In comparison, NCU-HOF1a showed a negligible increase in fluorescence intensity upon treatment with C_2_H_2_ or C_2_H_6_ gas, emphasizing its exceptional selectivity for C_2_H_4_ adsorption (Fig. S21†). Variable-temperature fluorescence tests revealed that NCU-HOF1a@C_2_H_4_ remains structurally stable under ambient conditions but readily releases adsorbed C_2_H_4_ during vacuum drying (Fig. S22†). This fluorescence enhancement can be repeated at least five times with C_2_H_4_ pressure ranging from 0 to 2 bar, indicating the excellent reusability of NCU-HOF1a for C_2_H_4_ uptake/release (Fig. S23a†). The unchanged PXRD pattern of NCU-HOF1a before and after C_2_H_4_ uptake demonstrated that the adsorbed C_2_H_4_ gas has no impact on its framework (Fig. S23b†). A summary of all the photophysical data of the as-prepared NCU-HOF1a@C_2_H_4_ is provided in Table S4.†

Fortunately, the C_2_H_4_-adsorbed NCU-HOF1a single crystal, named NCU-HOF1a@1C_2_H_4_, was obtained by exposing it to a C_2_H_4_ atmosphere at 1 bar and 273 K for approximately 24 hours (Table S1 and Fig. S24 and S25†). With the SCXRD results of NCU-HOF1a@1C_2_H_4_, the C_2_H_4_-induced fluorescence enhancement behavior of NCU-HOF1a can be further elucidated. Although the location and configuration of the C_2_H_4_ molecules within the pores are disordered, two possible modes of C_2_H_4_ molecules residing in the NCU-HOF1a framework were observed. In Fig. 5d, the C_2_H_4_ molecules were trapped between multiple Np synthons in NCU-HOF1a@1C_2_H_4_, forming weak C–H⋯O interactions (3.17–3.54 Å) in the type-I cavity, while forming moderate edge-to-face C–H⋯π interactions (2.64–3.46 Å) in the type-II cavity. These multiple intermolecular interactions between C_2_H_4_ molecules and Np synthons were also confirmed by the Hirshfeld surface analysis of the C_2_H_4_ molecule within the framework (Fig. S26†), further supporting the crucial role of Np synthons in trapping C_2_H_4_ molecules. Moreover, the NCU-HOF1a@1.75C_2_H_4_ single crystal was then obtained by raising the C_2_H_4_ pressure to 2 bar (Table S1†). The SCXRD analysis revealed a significant increase in the number of intermolecular interactions between C_2_H_4_ molecules and Np synthons, accompanied by a decrease in the shortest distance of these intermolecular interactions (Fig. S27 and S28†). This is mainly ascribed to the 1.75 times increase of C_2_H_4_ uptake in the NCU-HOF1a@1.75C_2_H_4_ compared to that of NCU-HOF1a@1C_2_H_4_. It should be noted that the unit cells of both C_2_H_4_-adsorbed NCU-HOF1a single crystals remain unchanged compared to NCU-HOF1a, indicating a gradual increase in the internal rigidity of the frameworks in the order of NCU-HOF1a@1.75C_2_H_4_ > NCU-HOF1a@1C_2_H_4_ > NCU-HOF1a. Precisely, the trapped C_2_H_4_ molecules within the pores of C_2_H_4_-adsorbed NCU-HOF1a restrict the rotation of the Np synthons, reducing the nonradiative decay pathways and resulting in the fluorescence turn-up of NCU-HOF1a. This is highly consistent with the experimental data, and all of these SCXRD analyses also strongly support the preferential adsorption of C_2_H_4_ over C_2_H_6_ on NCU-HOF1a under ambient conditions.

## Conclusions

In summary, we have demonstrated the facile self-correction of the hydrogen-bonding patterns in 3D NCU-HOFs, self-assembled from molecular units composed of peripheral Np hydrogen-bonded synthons and central bicarbazoles. The as-synthesized metastable NCU-HOF1 has an 11-fold interpenetrated dia topology with a quarter of the Np units exhibiting monomeric N–H⋯O interactions between adjacent Np link sites. These mismatched hydrogen bond patterns readily undergo an inconceivable “cross-door” rotation of nearly 180° upon desolvation, resulting in fully dimeric interactions. Consequently, the activated NCU-HOF1a becomes permanently porous with a BET surface area of 538 m^2^ g^−1^ and excellent robustness in highly polar solvents, strong acid, or alkaline aqueous solutions. Moreover, NCU-HOF1a demonstrates biased adsorption of C_2_H_4_ over C_2_H_6_ due to multiple C–H⋯O/π interactions between Np synthons and C_2_H_4_ guests in the contracted cavities. These host–guest interactions enhance the intramolecular rigidity of NCU-HOF1a, enabling efficient “turn-up” fluorescent sensing of C_2_H_4_. This work contributes to our understanding of self-correction dynamics in frameworks by nanoconfinement and their influence on the subsequent features and applications of organic porous materials.

## Author contributions

H. W. proposed the research direction and guided the project. G. X. and C. Z. designed and performed the materials synthesis and characterization. X. X., F. Y., and Y. Y. took part in the discussion of experimental data and gave useful suggestions. H. W., G. X., C. Z., and X. X. drafted the manuscript. All authors discussed the results and approved the final version of the manuscript.

## Conflicts of interest

There are no conflicts to declare.

## Supplementary Material

SC-015-D4SC02751E-s001

SC-015-D4SC02751E-s002

## Data Availability

The data are available from the corresponding author on reasonable request.

## References

[cit1] Lin R.-B., He Y. B., Li P., Wang H. L., Zhou W., Chen B. L. (2019). Chem. Soc. Rev..

[cit2] Lin Z.-J., Mahammed S. A. R., Liu T.-F., Cao R. (2022). ACS Cent. Sci..

[cit3] Zhang Z. J., Ye Y. X., Xiang S. C., Chen B. L. (2022). Acc. Chem. Res..

[cit4] Cai S. Z., An Z. F., Huang W. (2022). Adv. Funct. Mater..

[cit5] Hisaki I., Xin C., Takahashi K., Nakamura T. (2019). Angew. Chem., Int. Ed..

[cit6] Amooghin A. E., Sanaeepur H., Ghomi M., Luque R., Garcia H., Chen B. L. (2024). Coord. Chem. Rev..

[cit7] Li J. T., Chen B. L. (2024). Chem. Sci..

[cit8] Yang Y. S., Li L. B., Lin R.-B., Ye Y. X., Yao Z. Z., Yang L., Xiang F. H., Chen S. M., Zhang Z. J., Xiang S. C., Chen B. L. (2021). Nat. Chem..

[cit9] Zhang X., Li L. B., Wang J.-X., Wen H.-M., Krishna R., Wu H., Zhou W., Chen Z.-N., Li B., Qian G. D., Chen B. L. (2020). J. Am. Chem. Soc..

[cit10] Jiang C. H., Wang J.-X., Liu D., Wu E. Y., Gu X.-W., Zhang X., Li B., Chen B. L., Qian G. D. (2024). Angew. Chem., Int. Ed..

[cit11] Pal S. C., Mukherjee D., Sahoo R., Mondal S., Das M. C. (2021). ACS Energy Lett..

[cit12] Song Z. Y., Miao L., Ruhlmann L., Lv Y. K., Li L. C., Gan L. H., Liu M. X. (2023). Angew. Chem., Int. Ed..

[cit13] Han B., Wang H. L., Wang C. M., Wu H., Zhou W., Chen B. L., Jiang J. Z. (2019). J. Am. Chem. Soc..

[cit14] Qin W. K., Si D. H., Yin Q., Gao X. Y., Huang Q. Q., Feng Y. N., Xie L., Zhang S., Huang X. S., Liu T. F., Cao R. (2022). Angew. Chem., Int. Ed..

[cit15] Yu D. Q., Zhang H. C., Liu Z. Q., Liu C., Du X. B., Ren J. S., Qu X. G. (2022). Angew. Chem., Int. Ed..

[cit16] Tang J. K., Liu J., Zheng Q. Z., Li W. T., Sheng J. H., Mao L. Q., Wang M. (2021). Angew. Chem., Int. Ed..

[cit17] Yu D. Q., Zhang H. C., Ren J. S., Qu X. G. (2023). Chem. Soc. Rev..

[cit18] Song X. Y., Wang Y., Wang C., Wang D., Zhuang G. W., Kirlikovali K. O., Li P., Farha O. K. (2022). J. Am. Chem. Soc..

[cit19] Lin R.-B., Chen B. L. (2022). Chem.

[cit20] Cui P., Grape E. S., Spackman P. R., Wu Y., Clowes R., Day G. M., Inge A. K., Little M. A., Cooper A. I. (2020). J. Am. Chem. Soc..

[cit21] Li Y.-L., Alexandrov E. V., Yin Q., Li L., Fang Z.-B., Yuan W. B., Proserpio D. M., Liu T.-F. (2020). J. Am. Chem. Soc..

[cit22] Vicent-Morales M., Esteve-Rochina M., Calbo J., Ortí E., Vitórica-Yrezábal I. J., Espallargas G. M. (2022). J. Am. Chem. Soc..

[cit23] Feng S., Shang Y. X., Wang Z. K., Kang Z. X., Wang R. M., Jiang J. Z., Fan L. L., Fan W. D., Liu Z. N., Kong G. D., Feng Y., Hu S. Q., Guo H. L., Sun D. F. (2020). Angew. Chem., Int. Ed..

[cit24] Wang H. L., Li B., Wu H., Hu T.-L., Yao Z. Z., Zhou W., Xiang S. C., Chen B. L. (2015). J. Am. Chem. Soc..

[cit25] Chen T.-H., Popov I., Kaveevivitchai W., Chuang Y.-C., Chen Y.-S., Daugulis O., Jacobson A. J., Miljanic O. S. (2014). Nat. Commun..

[cit26] Hashim M. I, Le H. T. M., Chen T.-H., Chen Y.-S., Daugulis O., Hsu C.-W., Jacobson A. J., Kaveevivitchai W., Liang X., Makarenko T., Miljanic O. S., Popovs I., Tran H. V., Wang X. Q., Wu C.-H., Wu J. I. (2018). J. Am. Chem. Soc..

[cit27] Yan W. Q., Yu X. P., Yan T., Wu D. F., Ning E. L., Qi Y., Han Y.-F., Li Q. W. (2017). Chem. Commun..

[cit28] Maurya A., Marvaniya K., Dobariya P., Mane M. V., Tothadi S., Patel K., Kushwaha S. (2023). Small.

[cit29] Pulido A., Chen L. J., Kaczorowski T., Holden D., Little M. A., Chong S. Y., Slater B. J., McMahon D. P., Bonillo B., Stackhouse, C. J., Stephenson A., Kane C. M., Clowes R., Hasell T., Cooper A. I., Day G. M. (2017). Nature.

[cit30] Mastalerz M., Oppel I. M. (2012). Angew. Chem., Int. Ed..

[cit31] Chen X.-Y., Cao L.-H., Bai X.-T., Cao X.-J. (2024). Chem. Eur. J..

[cit32] Karmakar A., Illathvalappil R., Anothumakkool B., Sen A., Samanta P., Desai A. V., Kurungot S., Ghosh S. K. (2016). Angew. Chem. Int. Ed..

[cit33] Sun Y. Y., Wei J., Fu Z. H., Zhang M. Y., Zhao S. G., Xu G., Li C. S., Zhang J., Zhou T. H. (2023). Adv. Mater..

[cit34] Desiraju G. R. (1995). Angew. Chem., Int. Ed. Engl..

[cit35] Little M. A., Cooper A. I. (2020). Adv. Funct. Mater..

[cit36] Wang B., Lin R.-B., Zhang Z. J., Xiang S. C., Chen B. L. (2020). J. Am. Chem. Soc..

[cit37] Zhu Q., Wei L., Zhao C., Qu H. X., Liu B. W., Fellowes T., Yang S. Y., Longcake A., Hall M. J., Probert M. R., Zhao Y. B., Cooper A. I., Little M. A. (2023). J. Am. Chem. Soc..

[cit38] Chen S. M., Ju Y., Yang Y. S., Xiang F. H., Yao Z. Z., Zhang H., Li Y. B., Zhang Y. F., Xiang S. C., Chen B. L., Zhang Z. J. (2024). Nat.Commun..

[cit39] Wang S. C., Zhang Q. S., Wang Z., Guan S. Q., Zhang X. D., Xiong X. H., Pan M. (2023). Angew. Chem., Int. Ed..

[cit40] Wang D., Zhao Y. (2023). Angew. Chem., Int. Ed..

[cit41] Jiang J. C., Zhao Y. B., Yaghi O. M. (2016). J. Am. Chem. Soc..

[cit42] Mohammed A. K., Raya J., Pandikassala A., Addicoat M. A., Gaber S., Aslam M., Ali L., Kurungot S., Shetty D. (2023). Angew. Chem. Int. Ed..

[cit43] Hashimoto T., Oketani R., Nobuoka M., Seki S., Hisaki I. (2023). Angew. Chem., Int. Ed..

[cit44] Yang X., Huang J., Gao S. Y., Zhao Y. Q., Huang T., Li H. F., Liu T. F., Yu Z. Y., Cao R. (2023). Adv. Mater..

[cit45] Hu F. L., Liu C. P., Wu M. Y., Pang J. D., Jiang F. L., Yuan D. Q., Hong M. C. (2017). Angew. Chem., Int. Ed..

[cit46] Rahman M. A., Dionne C. J., Giri A. (2022). Nano Lett..

[cit47] Gao J. K., Cai Y. L., Qian X. F., Liu P. X., Wu H., Zhou W., Liu D.-X., Li L. B., Lin R.-B., Chen B. L. (2021). Angew. Chem., Int. Ed..

[cit48] Li P. H., Li P., Ryder M. R., Liu Z. C., Stern C. L., Farha O. K., Stoddart J. F. (2019). Angew. Chem., Int. Ed..

[cit49] Ji Q., Takahashi K., Noro S.-I., Ishigaki Y., Kokado K., Nakamura T., Hisaki I. (2021). Cryst. Growth Des..

[cit50] Zhou Y. Z., Chen C., Krishna R., Ji Z. Y., Yuan D. Q., Wu M. Y. (2023). Angew. Chem., Int. Ed..

[cit51] Ding X. J., Liu Z. Y., Zhang Y. S., Ye G., Jia J. F., Chen J. (2022). Angew. Chem., Int. Ed..

[cit52] Yu B. Q., Geng S. B., Wang H. L., Zhou W., Zhang Z. J., Chen B. L., Jiang J. Z. (2021). Angew. Chem., Int. Ed..

[cit53] Lv Y., Liang J., Xiong Z., Zhang H., Li D., Yang X., Xiang S., Zhang Z. (2023). –. Chem.–Eur. J..

[cit54] Cai Y. L., Gao J. K., Li J.-H., Liu P. X., Zheng Y. C., Zhou W., Wu H., Li L. B., Lin R.-B., Chen B. L. (2023). Angew. Chem., Int. Ed..

[cit55] Wang Y., Ma K., Bai J. Q., Xu T., Han W. D., Wang C., Chen Z. X., Kirlikovali K. O., Li P., Xiao J. S., Farha O. K. (2022). Angew. Chem., Int. Ed..

[cit56] Suzuki Y., Tohnai N., Saeki A., Hisaki I. (2020). Chem. Commun..

[cit57] Ye G. G., Wang Y. G., Hong L. M., Yu F. Q., Xia G. M., Wang H. M. (2021). J. Mater. Chem. C..

[cit58] Zhou C. L., Wang M. D., Guo W. H., Ye, G. G., Wang Y. G., Yang Y., Xia G. M., Wang H. M. (2023). Dyes Pigm..

[cit59] Spek A. L. (2003). J. Appl. Crystallogr..

[cit60] Yang W., Wang J. W., Wang H. L., Bao Z. B., Zhao J. C.-G., Chen B. L. (2017). Cryst. Growth Des..

[cit61] Sengupta D., Melix P., Bose S., Duncan J., Wang X. J., Mian M. R., Kirlikovali K. O., Joodaki F., Islamoglu T., Yildirim T., Snurr R. Q., Farha, O. K. (2023). J. Am. Chem. Soc..

