# Supplementary material for: Self-correcting mismatches in metastable hydrogen-bonded organic frameworks with an 11-fold interpenetrated array
Source: Chem Sci. 2024 Aug 6;15(35):14336–43. doi: 10.1039/d4sc02751e (PMC11325195; doi:10.1039/d4sc02751e)
Supplement: SC-015-D4SC02751E-s001 [file SC-015-D4SC02751E-s001.pdf]

Supplementary Information (SI) for Chemical Science.
This journal is © The Royal Society of Chemistry 2024

Electronic Supplementary Information

Self-Correcting Mismatches in Metastable Hydrogen-Bonded Organic Frameworks with 11-fold Interpenetrated Array

Guomin Xia[a†], Chunlei Zhou[a†], Xingliang Xiao[a], Yang Yang[a], Fuqing Yu[a], Hongming Wang[a*]

[a] Institute for Advanced Study and College of Chemistry, Nanchang University, 999 Xuefu Avenue, Nanchang 330031, P. R. China

[†] These authors contributed equally to this work.

[*]Corresponding author. E-mail: hongmingwang@ncu.edu.cn.

Materials and Methods

^1H and ^{13}C NMR spectra were measured using a Bruker AVANCE 400 spectrometer in dimethyl sulfoxide (DMSO)-d_6 or chloroform ($CDCl_3$)-d_3 using tetramethylsilane as an internal standard. UV-visible absorption spectra were recorded using a Lambda 750 spectrophotometer. Photoluminescence (PL) spectra were recorded using a Horiba FluoroMax-4 luminescence spectrometer. Absolute Φ_{PL} values were determined using a Horiba FL-3018 integrating sphere. Fluorescence lifetime measurements were performed on a Horiba FluoreCube spectrofluorometer system using a UV diode laser (NanoLED; 367 nm) for excitation. Scanning electron microscopy (SEM) images were collected on a Hitachi S-4300 instrument. Mass spectra were obtained using a Trip TOFTM 5600 mass spectrometer and SCIEX X500R QTOF mass spectrometer. Thermogravimetric analysis (TGA) was carried out on a Dimand TG/DTA instrument at a heating rate of 10 °C min^{-1} under an N_2 atmosphere. The IR analysis was performed with a BRUKER TENSOR II FTIR spectrometer equipped with a crucible for variable-temperature tests. Powder X-ray diffraction (PXRD) data were collected using a SmartLab9KW X-ray diffractometer in the parallel beam geometry employing CuKα radiation at 40 kV and 30 mA. Diffraction data were collected in the 2θ range from 2.5° to 30° at a scan time of 1.54 s per step and a 2θ step increment of 0.02°.

Syntheses

Np: The compound is accessed by commercial ways. Then, the crude product was further purification by recrystallized in acetic ester to yield pale green power. ^1H NMR (400 MHz, DMSO-d_6) δ: 10.79 (s, 1H), 8.19 (t, J = 10.11 Hz, 1H), 8.01 (t, J = 9.70 Hz, 1H), 7.87-7.78 (m, 1H), 7.60 (d, J = 8.44 Hz, 1H), 7.50 (d, J = 14.18, 5.79 Hz, 1H), 6.99 (t, J = 6.49 Hz, 1H). ^{13}C NMR (100 MHz, DMSO-d6) δ: 169.27, 138.56, 131.36, 129.54, 129.49, 129.39, 127.34, 126.06, 124.16, 119.89, 106.55. HRMS (ESI) m/z: $[M + H]^+$ calculated for $[M]^+$, 170.0600; found, 170.0577.

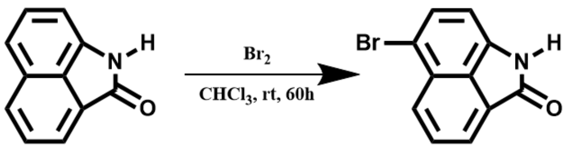

Np-Br: Np-Br was prepared according to the literature procedure. Np (5.01 g, 29.1 mmol) and chloroform (120 mL) was added to a 250ml round flask, then bromine (7.10 g, 44.4 mmol) was added drop- by-drop to the reaction system

and stirred at 298K for 60 h. Then saturated sodium thiosulfate aqueous solution (100 mL) was used to quenching the excessive amounts of bromine. The resulting precipitate was filtered and washed with deionized water and dried under high vacuum at 50° for 24 h to give crude product as brownish yellow powder. The crude product was purified by column chromatography (V:V petroleum ether:ethyl acetate = 3:1) to give pure Np-Br as pale yellow powder (5.85 g, 23.58 mmol, 81%). ^{1}H NMR (400 MHz, DMSO-d_6) δ: 10.91 (s, 1H), 8.19-8.04 (m, 2H), 7.93 (d, J = 8.12, 7.11 Hz, 1H), 7.76 (d, J = 7.54 Hz, 1H), 7.64-7.54 (m, 1H), 6.93 (t, J = 8.25 Hz, 1H). ^{13}C NMR (100 MHz, DMSO-d6) δ: 168.61, 138.55, 132.31, 130.88, 129.98, 128.81, 127.79, 127.11, 125.26, 112.31, 107.91. HRMS (ESI) m/z: $[M + H]^+$ calculated for M^+, 247.9706; found, 247.9711.

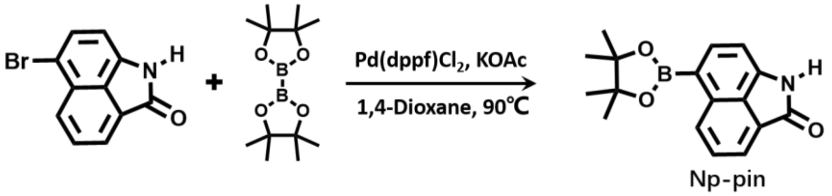

Np-pin: A 100 mL round-bottom flask was charged with Np-Br (2.48 g, 10.0 mmol), bis(pinacolato)diboron (3.05 g, 12.0 mmol) and potassium acetate (3.52 g, 36 mmol) dissolved in 80 mL 1,4-Dioxane. Then Pd(dppf)Cl_2 (500 mg, 0.68 mmol) was added to the solution under a nitrogen atmosphere. After the addition, the reaction was stirred at 90 ℃ for 24 h. Then cooling and removing most solvent of the resulting mixture and diluted with CH_2Cl_2 (300 mL), following with deionized water (600 mL) and saturated NaCl solution (200 mL) to wash it. The organic solvent was dried over Na_2SO_4 and removed in vacuo to get crude product as brown powder and separated on a silica gel column using CH_2Cl_2/ EA as eluent (V:V CH_2Cl_2:EA = 50 : 1) to give the product Np-pin (1.95g, 6.60mmol, 66%). ^{1}H NMR (400 MHz, DMSO-d_6) δ: 10.90 (s, 1H), 8.64 (d, J = 8.15 Hz, 1H), 8.18-8.10 (m, 1H), 7.90 (d, J = 15.22, 12.02, 6.96 Hz, 4H), 7.61-7.53 (m, 1H), 6.99 (d, J = 7.14 Hz, 1H), 4.04-3.96 (m, 1H), 3.96-3.88 (m, 1H), 3.59-3.51 (m, 1H), 3.32 (s, 1H), 2.56-2.44 (m, 1H), 1.98 (s, 1H), 1.50 (s, 1H), 1.38 (d, J = 28.11 Hz, 15H), 1.12 (d, J = 52.20 Hz, 1H), -0.02 (s, 1H). ^{13}C NMR (100 MHz, DMSO-d6) δ: 169.34, 141.66, 139.53, 132.73, 132.34, 129.72, 127.16, 125.96, 124.06, 106.12, 82.89. HRMS (ESI) m/z: $[M+H]^+$ calculated for M^+, 296.1453; found, 296.2567.

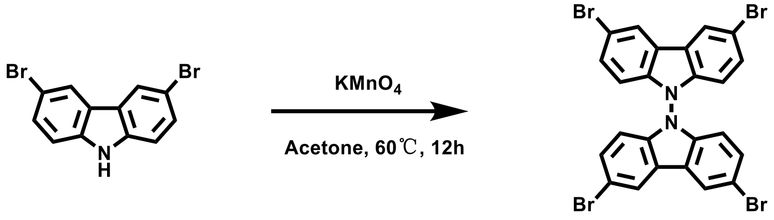

BC-4Br: According to the literature method, 3,6-dibromocarbazole (1.63 g, 5.0 mmol) and acetone (30 mL) were added to a 50 mL round-bottom flask and stirred until dissolved. Under ice bath conditions, potassium permanganate (1.98 g, 12.5 mmol) was slowly added, and the reaction mixture was refluxed at 60 °C for 12 hours with the reaction progress monitored by TLC. After completion of the reaction, the mixture was cooled to 298K. Acetone was removed under rotary evaporation, followed by the addition of 30 mL of $CHCl_3$. The mixture was filtered to remove any solid residues. The filtrate was washed with sodium dithionite solution and saturated brine, then dried with anhydrous magnesium sulfate. The crude product was obtained by vacuum concentration, and further purification was achieved by column chromatography (CH_2Cl_2: n-hexane = 3:1) to yield white powder BC-4Br (1.18 g, 73% yield). ^{1}H NMR (400 MHz, $CDCl_3$-d_1) δ 8.27 (s, 1H), 7.46 (d, J = 8.6 Hz, 1H), 6.74 (d, J = 9.1 Hz, 1H). ^{13}C NMR (101 MHz, $CDCl_3$-d_1) δ 138.51, 130.39, 123.93, 122.67, 115.00, 110.52. HRMS (ESI) m/z: $[M-H]^-$ calculated for M^-, 642.7661; found, 642.7674.

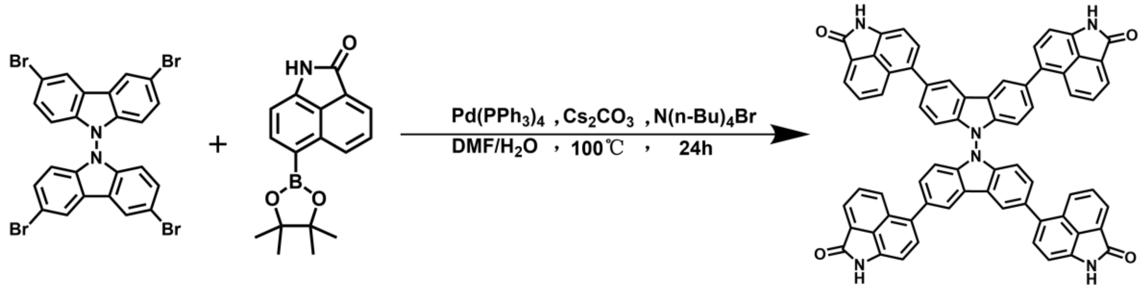

BC-4Np: DMF (80 mL), Np-pin (1.27 g, 4.3 mmol), BC-4Br (0.62 g, 0.96 mmol), cesium carbonate (3.74 g, 11.5 mmol), and tetrabutylammonium bromide (20 mg) were added to a 120 mL pressure vessel, followed by the addition of deionized water (8 mL). The vessel was subjected to vacuum and nitrogen purging cycles three times. Subsequently, tetrakis(triphenylphosphine)palladium (600 mg, 0.6 mmol) was added to the reaction mixture, which was stirred at 100 ℃ for 24 hours, with the reaction progress monitored by TLC. After completion of the reaction, the mixture was filtered, and the crude product was washed sequentially with 50 mL of dichloromethane, 50 mL of ethanol, and 50 mL of deionized water. The resulting crude product was then subjected to recrystallization from DMF/acetone, yielding yellow-green pure BC-4Np (766 mg, 80% yield). ^{1}H NMR (400 MHz, DMSO-d_6) δ 10.85 (s, 1H), 8.73 (s, 1H), 8.24 (d, J = 8.3 Hz, 1H), 8.04 (d, J = 6.9 Hz, 1H), 7.79 (t, J = 7.6 Hz, 1H), 7.62 (d, J = 8.4 Hz, 1H), 7.55 (d, J = 7.2 Hz, 1H), 7.16 (d, J = 8.3 Hz, 1H), 7.07 (d, J = 7.3 Hz, 1H). ^{13}C NMR (101 MHz, DMSO-d_6) δ 169.27, 139.63, 137.82, 133.28, 132.89, 130.26, 129.88, 129.79, 129.48, 127.99, 127.67, 126.32, 124.28, 123.13, 122.64, 109.48, 106.90. HRMS m/z: $[M+H]^+$ calculated for M^+, 1001.2871; found, 1001.2117.

Preparation of Single Crystals

NCU-HOF1: The single crystals of NCU-HOF1 were obtained using a gas-liquid diffusion method. Initially, 10 mg of BC-4Np was completely dissolved in 2 mL of DMF. The solution was then subjected to filtration through a membrane to remove insoluble impurities. The filtrate was subsequently transferred into a glass vial with a volume of 10 mL. The vial was placed at the bottom of a 100 mL beaker containing anhydrous sodium sulfate. Following this, a specific quantity of acetone was added, and the beaker was sealed and positioned in an undisturbed environment at a controlled ambient temperature of 25 ℃. After approximately 10 days, colorless to pale yellow block-shaped single crystals of NCU-HOF1 were obtained.

NCU-HOF1a: The single crystal of NCU-HOF1 was immersed in anhydrous acetone for a solvent exchange process lasting 12 hours. Subsequently, the acetone was removed, and an equal volume of fresh anhydrous acetone was added. This process was repeated approximately five times. Following the solvent exchange, the single crystal was subjected to ultra-high vacuum treatment at 40 ℃ for 72 hours. Vacuum was re-established every 24 hours during this period. As a result of these procedures, the activated single crystal NCU-HOF1a was obtained from NCU-HOF1.

NCU-HOF1a@1C_2H_4: The vial containing NCU-HOF1a single crystal was placed inside a 120 mL pressure-resistant flask. The flask was then subjected to vacuum using an oil pump under heating conditions at 100 ℃ to remove residual moisture and gases from the pores. The system was vacuum-dried for 1 hour. Subsequently, the heating was turned off, and the system was allowed to cool. Afterward, pure ethylene was introduced into the system and evacuated three times in a cyclical manner to ensure thorough gas exchange. Ethylene was then introduced to maintain a pressure of 1 bar within the system. The system was sealed and placed in a mixture of ice and water to achieve thermal equilibrium for adsorption. After 12 hours, NCU-HOF1a@1C_2H_4 single crystals, with ethylene gas adsorbed in their pores, were obtained. The single crystals were further subjected to sealed-tube testing at 150 K.

NCU-HOF1a@1.75C_2H_4: The preparation process of NCU-HOF1a@1.75C_2H_4 is similar to that of NCU-HOF1a@1C_2H_4, with the only difference being the control of the amount of ethylene introduced, resulting in a system pressure of 2 bar.

Single-crystal X-ray diffraction studies

Data collection and structural analysis of the crystals were performed using a Bruker SMART APEX-II single-crystal

diffractometer equipped with graphite monochromatic CuKα radiation. The structures were solved with the Shelxl-97 structure solution program using intrinsic phasing and refined with the Shelxl-97 refinement package using least-squares minimization through Olex2 software.

For NCU-HOF1 and NCU-HOF1a, the cultured single crystals were sealed in suitable tubes and tested at 150 K. While The process for NCU-HOF1a@1C_2H_4 was as follows: The glass bottle containing the NCU-HOF1a single crystal was placed in a 120 ml pressure-resistant bottle. An oil pump was used under heating at 100°C to vacuum remove residual moisture and impurities from the pore channels, with the sample vacuum-dried for 1 hour. The heating was then turned off to allow the system to cool. Pure C_2H_4 was repeatedly filled and evacuated three times, followed by filling with C_2H_4 to maintain system pressure at 1 bar. The system was sealed and placed in an ice-water mixture for constant-temperature equilibrium adsorption. After 12 hours, the NCU-HOF1a@1C_2H_4 single crystal, now adsorbed with ethylene gas in the pore channels, was sealed and tested at 150 K. The same process was followed for NCU-HOF1a@1.75C_2H_4 unless the system pressure was maintain at 2 bar.

Preparation of microcrystals

NCU-HOF1: To synthesize NCU-HOF1 microcrystals, 800 mg of BC-4Np was completely dissolved in a mixed solvent (80 mL DMF and 8 mL deionized water) under heating conditions at 100 ℃. The solution was quickly filtered while hot to remove insoluble impurities. The filtrate was then maintained at a constant temperature of 90 ℃ with continuous stirring, allowing the gradual formation and aging of microcrystals. Filtration under reduced pressure resulted in the assembly of well-ordered NCU-HOF1 microcrystals.NCU-HOF1a: Due to the relatively poor crystal stability of NCU-HOF1, newly prepared NCU-HOF1 microcrystals needed to be promptly activated through vacuum drying. To avoid rapid solvent removal from the pores, which could destabilize the crystals, a programmed vacuum heating method was employed. Initially, the NCU-HOF1 microcrystals were subjected to vacuum drying at 30 ℃ for 3 hours. Subsequently, the temperature was increased by 10 ℃ every 3 hours until reaching a constant temperature of 100 ℃ for another 3 hours. This process resulted in the activation of NCU-HOF1a microcrystals while preserving their morphology effectively.

Fitting of experimental data on pure component isotherms

The single-component adsorption isotherms for C_2H_4 and C_2H_6 in NCU-HOF1a were determined by fitting the adsorption isotherms at 298 K, 313 K and 328 K for the respective adsorbates to the dual-site Langmuir-Freundlich (DSLF) equation. The DSLF equation was given by:

$$q = q_{A,sat}\, b_A p^{n_A}/(1+b_A p^{n_A})+q_{B,sat}\, b_B p^{n_B}/(1+b_B p^{n_B}) \quad (1)$$

where q is the uptake (in mmol/g), P is the pressure (in kPa), $q_{A,sat}$ and $q_{B,sat}$ are the saturation uptakes (in mmol/g) for sites 1 and 2, b_A and b_B are the affinity coefficients (in /kPa) for sites 1 and 2, and n_A and n_B represent the deviations from the ideal homogeneous surface. The single-component adsorption isotherms for C_2H_4 and C_2H_6 were fitted by the above form of DSLF equation. The fitting parameters were displayed in Table S3.

Calculation of isosteric heat of adsorption (Q_{st})

The experimental adsorption enthalpy (Q_{st}) was applied to evaluate the binding

strength between adsorbent and adsorbate, defined as:

$$Q_{st} = RT^2 (\partial \ln p / \partial T)_P \quad (2)$$

The isosteric heat of adsorption, Q_{st} is determined using the pure component isotherm fits using the Clausius-Clapeyron equation, where Q_{st} (kJ mol^{-1}) is the isosteric heat of adsorption, T (K) is the temperature, p (kPa) is the pressure, and R is the gas constant.

Computational study

Computational methods: All calculations were performed using Gaussian 09 package1. Theoretical calculation for the geometrical optimizations was performed by density functional theory (DFT) using B3LYPmethod.2,3 The 6-31G (d,p) basis sets were employed for all atoms. We ascertained that all the transition states have only one imaginary frequency through vibrational analysis.

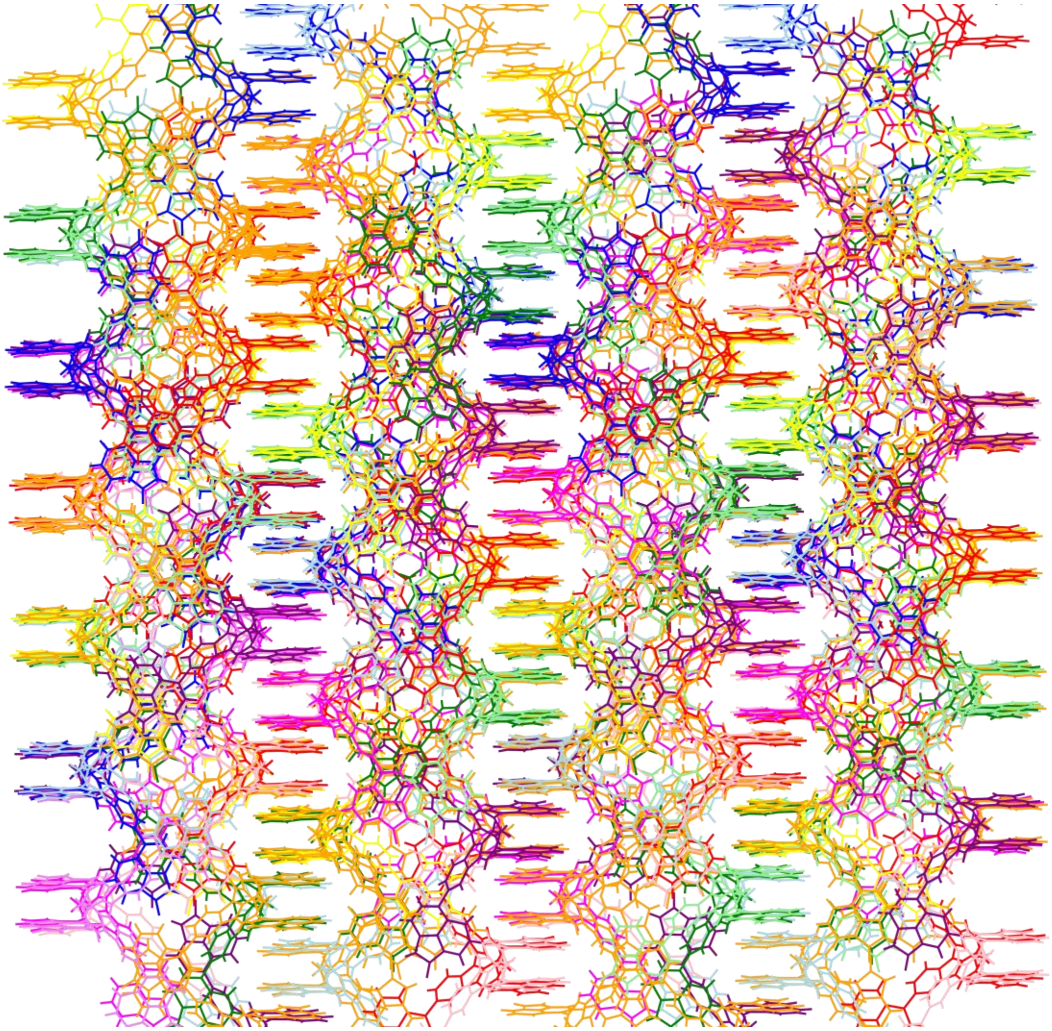

Figure S1. Schematic diagram of structure analysis for NCU-HOF1.

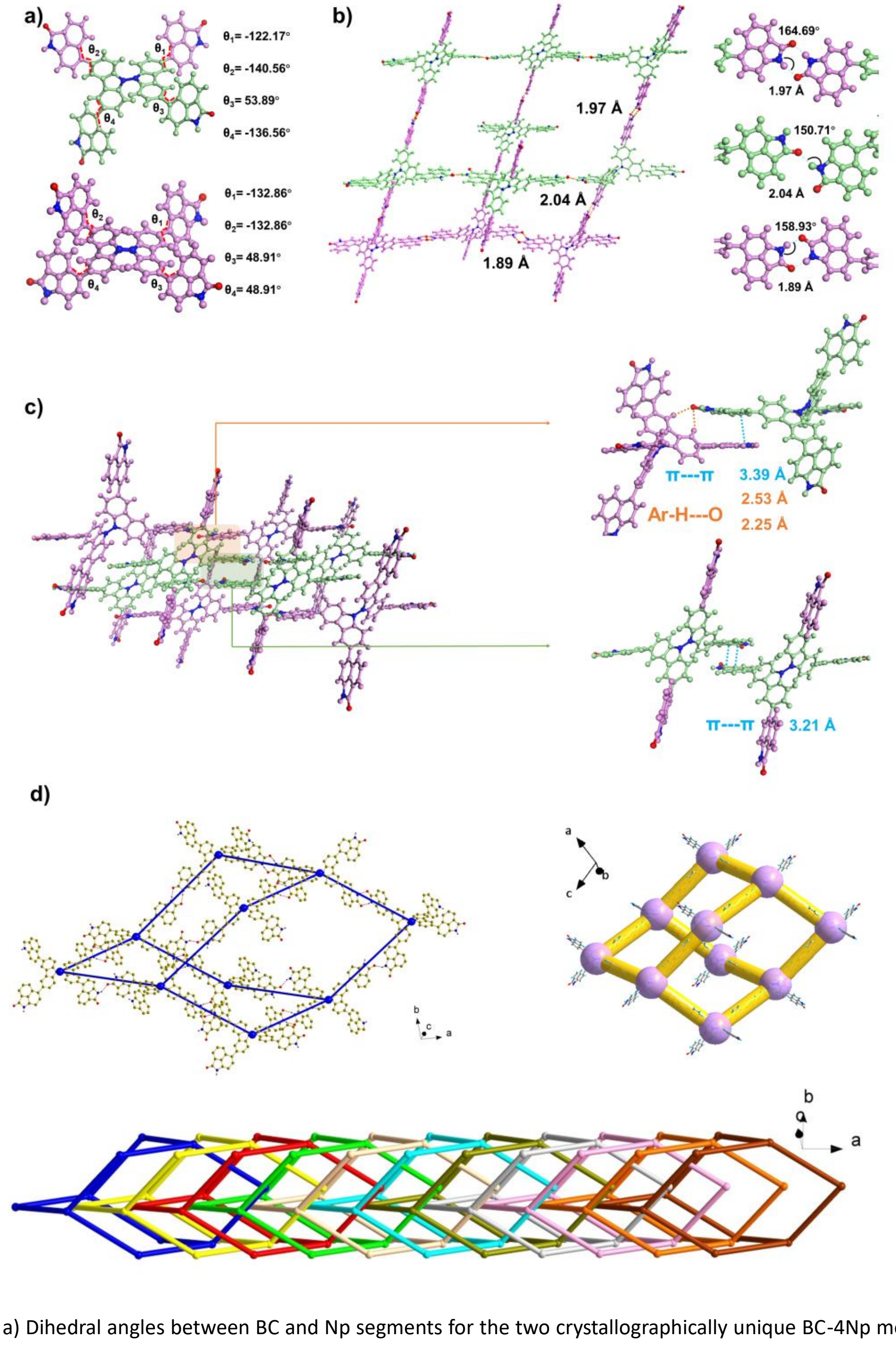

Figure S2. a) Dihedral angles between BC and Np segments for the two crystallographically unique BC-4Np molecules. b) The distance and angle of alternately monomeric and dimeric N-H···O hydrogen bonds between adjacent BC-4Np in *dia* topology. c) The molecular stacking between adjacent BC-4Np molecules. d) The *dia* topology and 11-fold interpenetrated topological frameworks of BC-4Np.

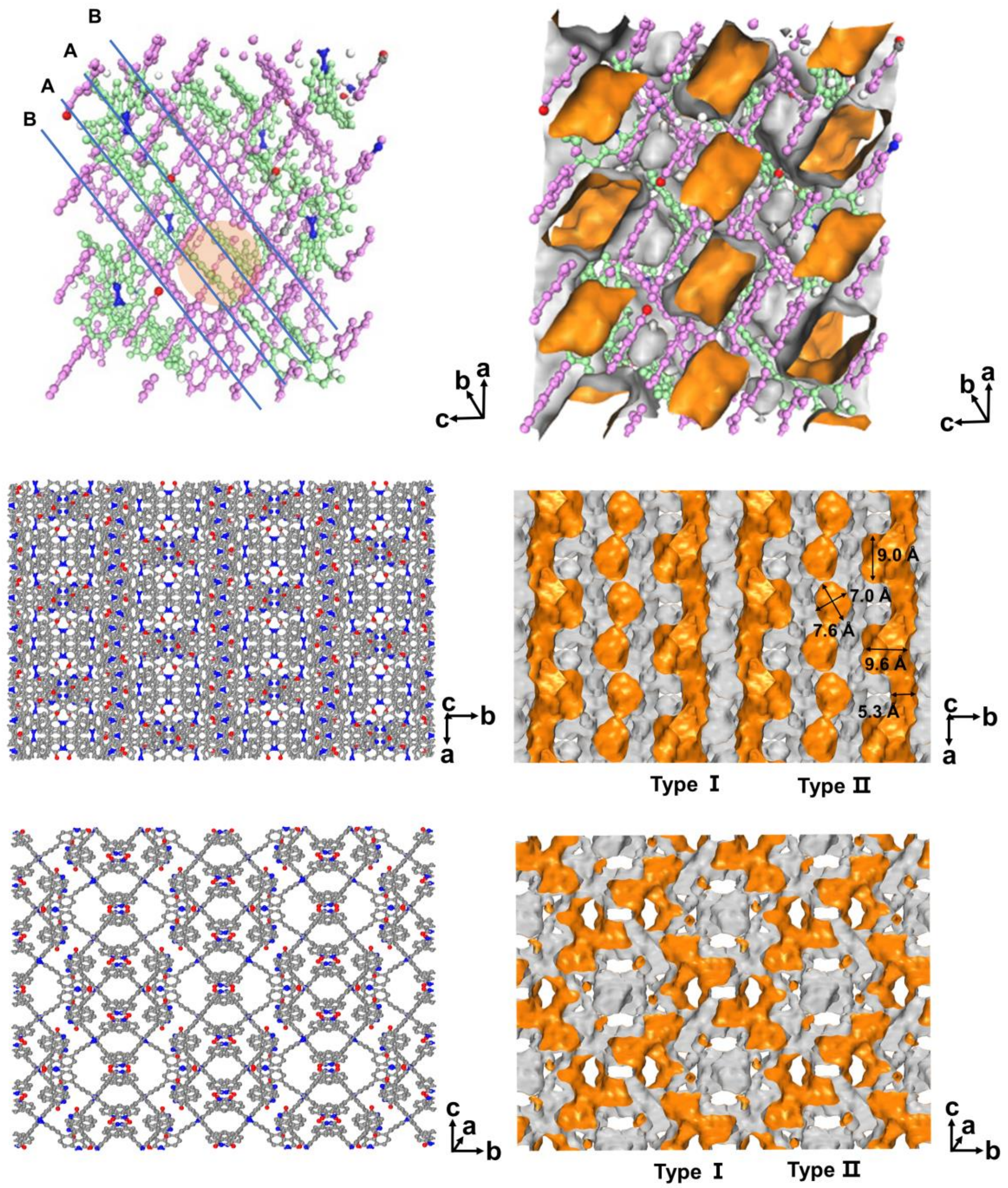

Figure S3. Framework with two pore channels in NCU-HOF1 from different perspectives.

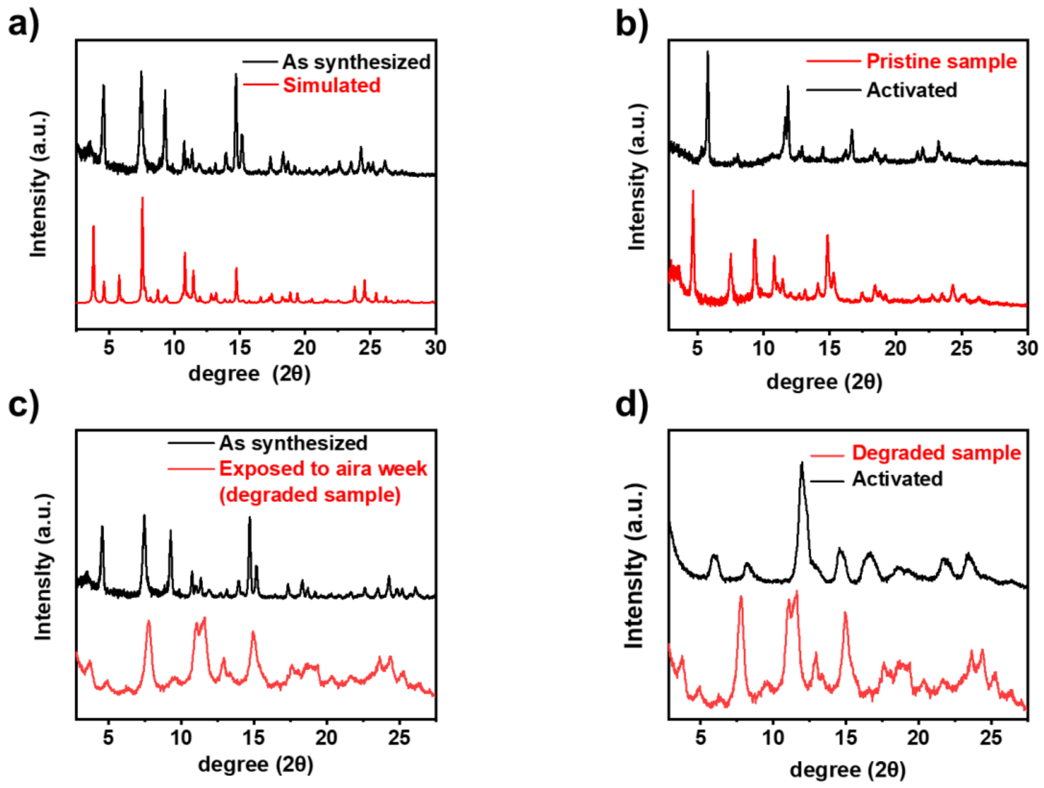

Figure S4. PXRD patterns before and after activation of NCU-HOF1.

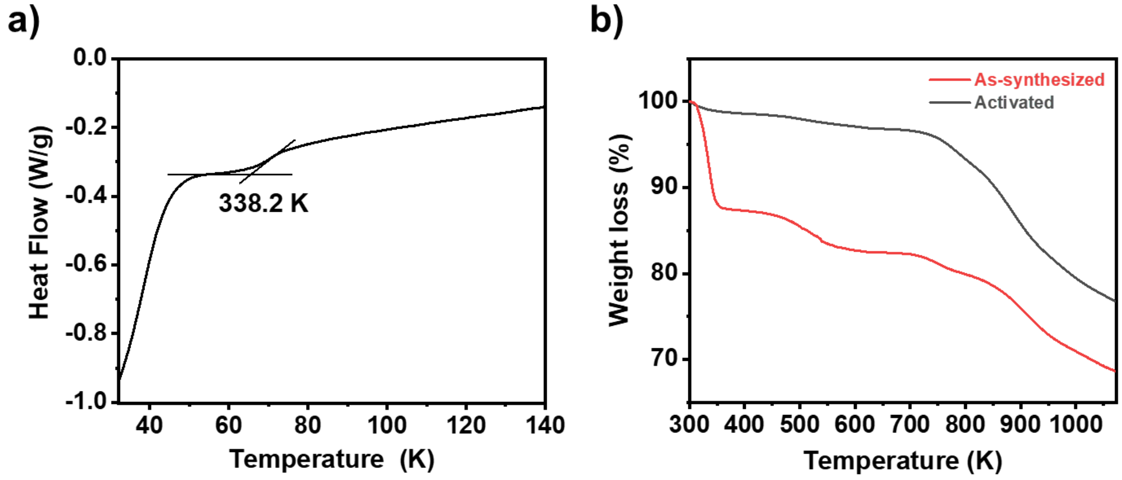

Figure S5. a) DSC curve of as-synthesized NCU-HOF1. b) TGA curves of NCU-HOF1 before and after activation.

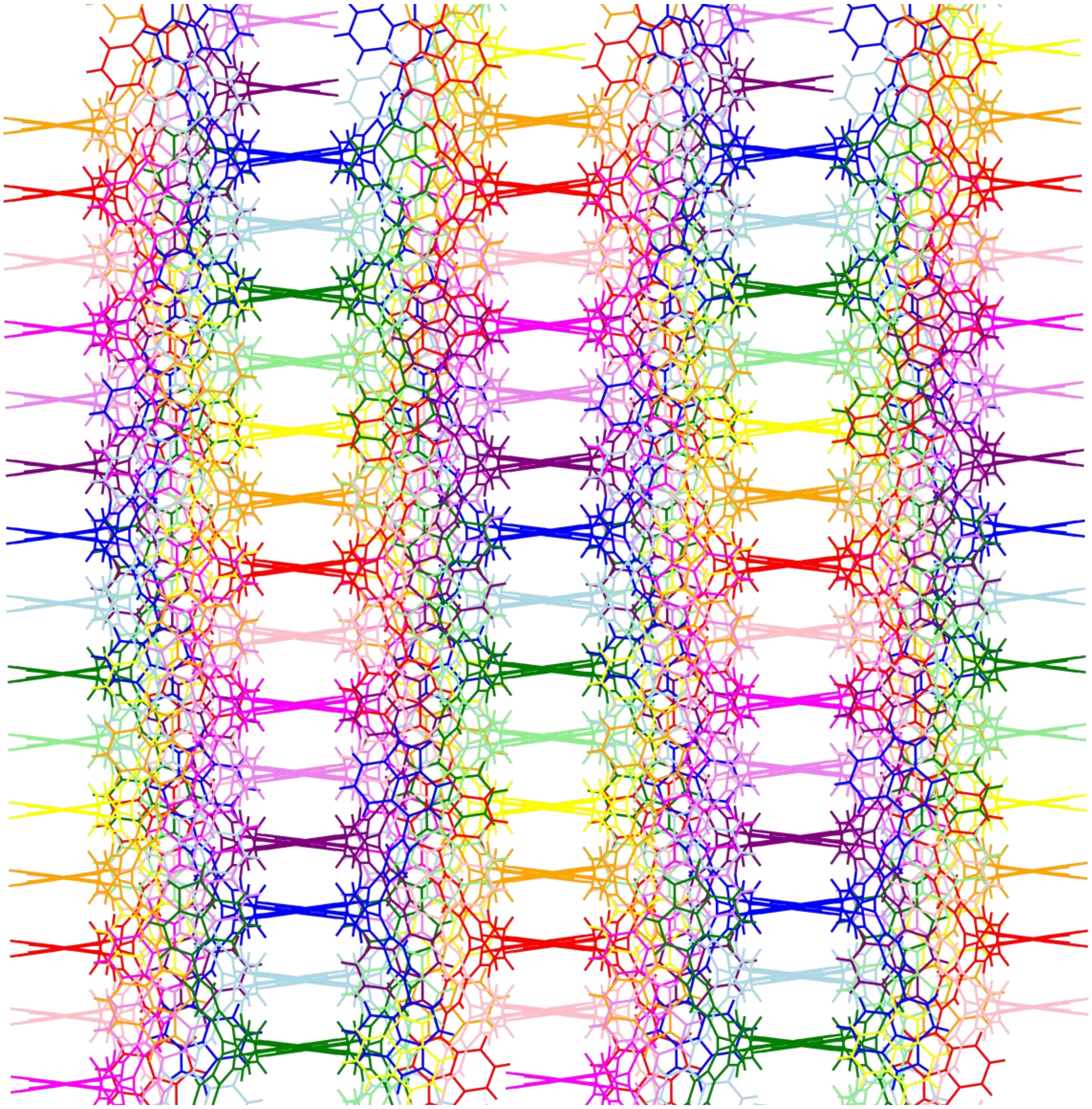

Figure S6. Schematic diagram of structure analysis for NCU-HOF1a.

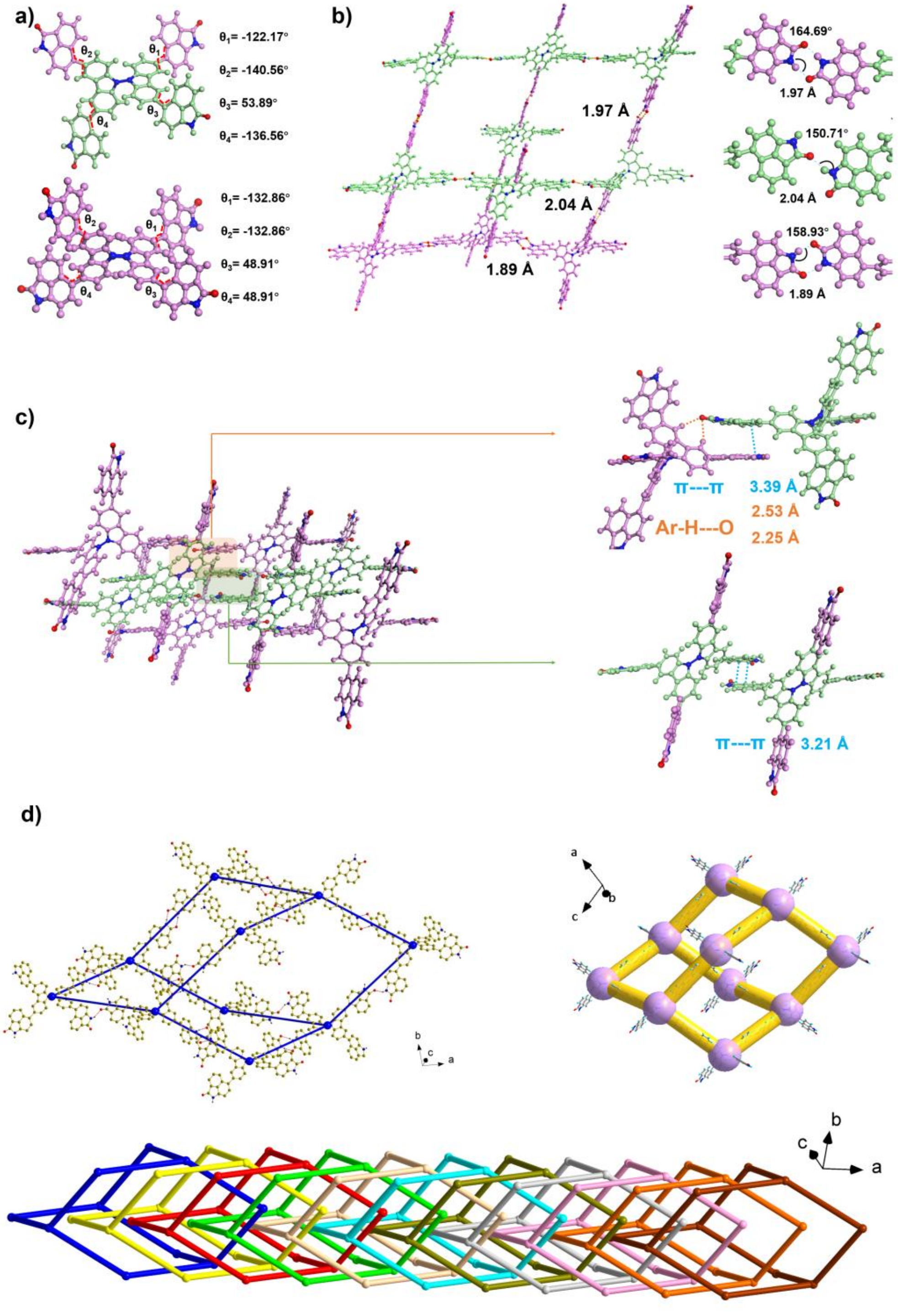

Figure S7. a) Dihedral angles between BC and Np segments. b) Distances and angles of alternately monomeric and dimeric N-H···O hydrogen bonds between adjacent BC-4Np molecules in dia topology. c) Molecular stacking between adjacent BC-4Np molecules. d) Dia topology and 11-fold interpenetrated topological frameworks of BC-4Np.

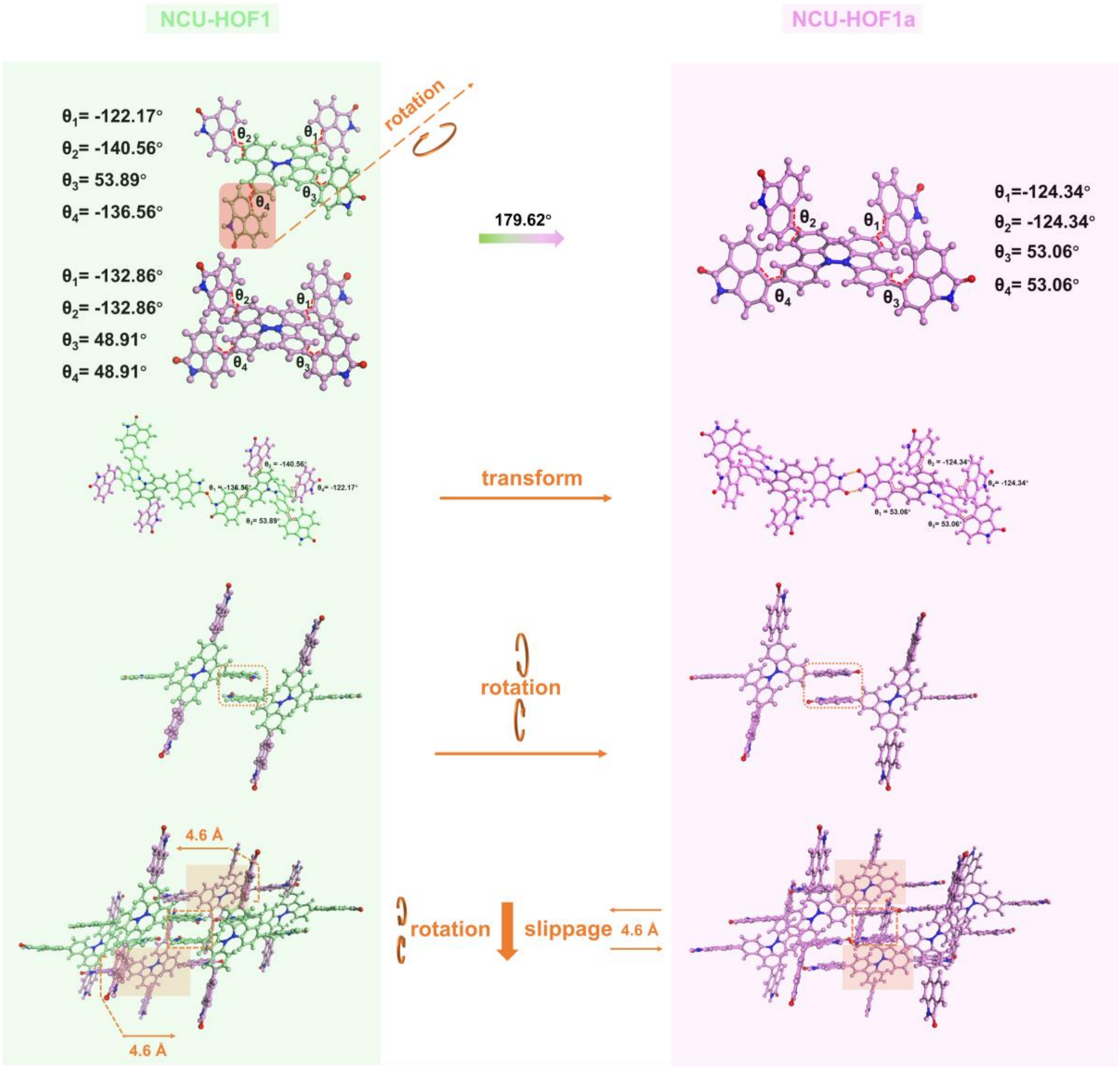

Figure S8. Structural analysis of in-situ nanoconfinement isomerization from NCU-HOF1 to NCU-HOF1a during desolvation.

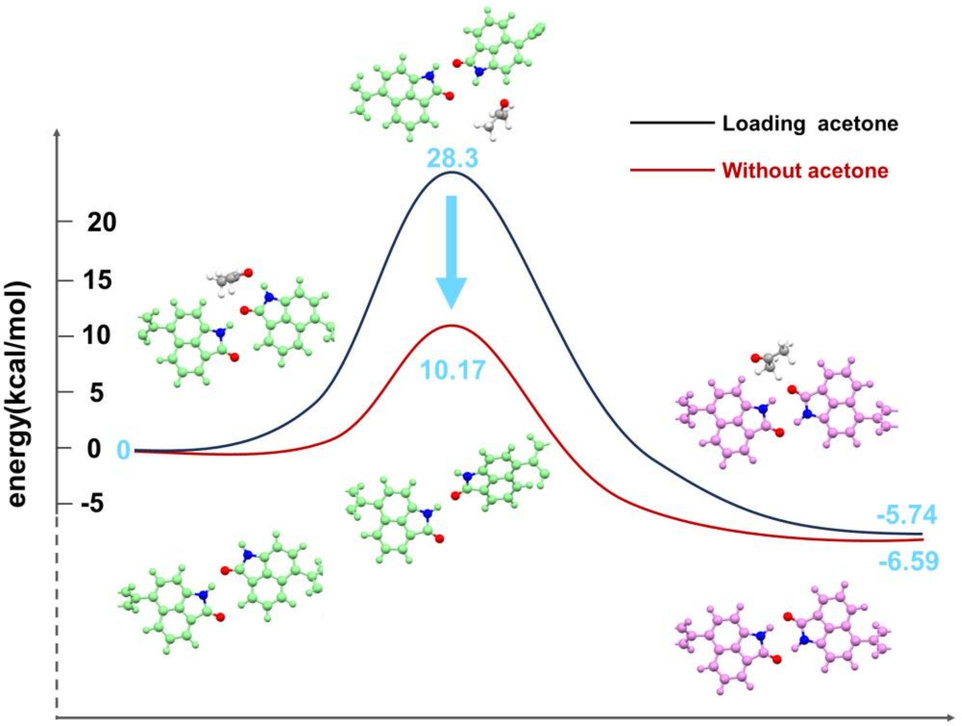

Figure S9. Energy profile of BC-4Np monomer to dimer formation in NCU-HOF1, with (black line) and without (red line) guest acetone molecules.

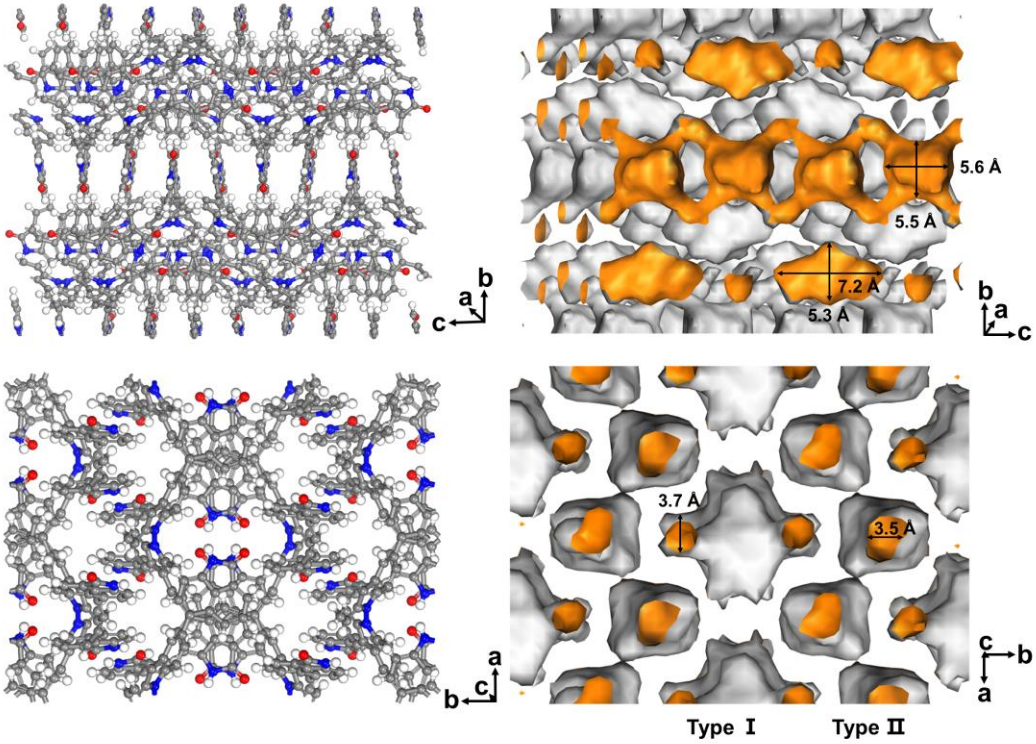

Figure S10. Framework of NCU-HOF1a with two pore channels shown from different perspectives.

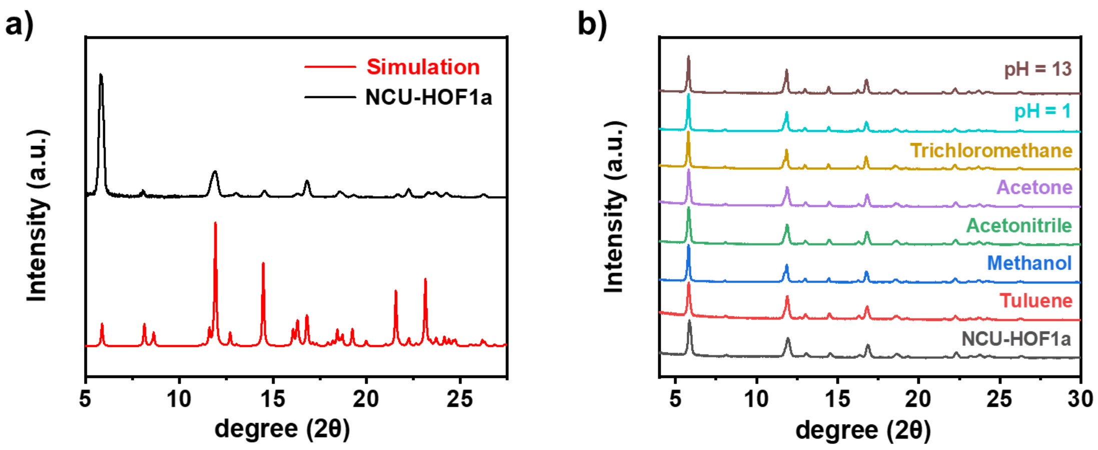

Figure S11. a) PXRD patterns of NCU-HOF1a and the corresponding crystals. b) PXRD patterns of NCU-HOF1a after one month of immersion in different solutions and aqueous solutions with varying pH.

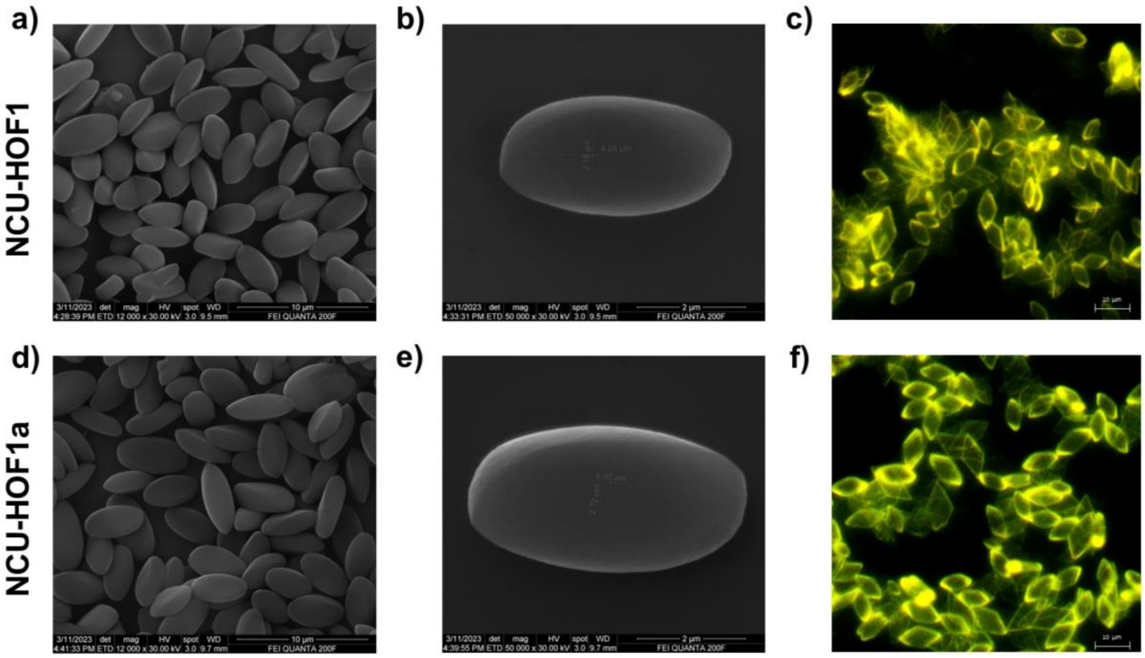

Figure S12. SEM images and fluorescence microscope photographs of NCU-HOF1 (top) and NCU-HOF1a (bottom).

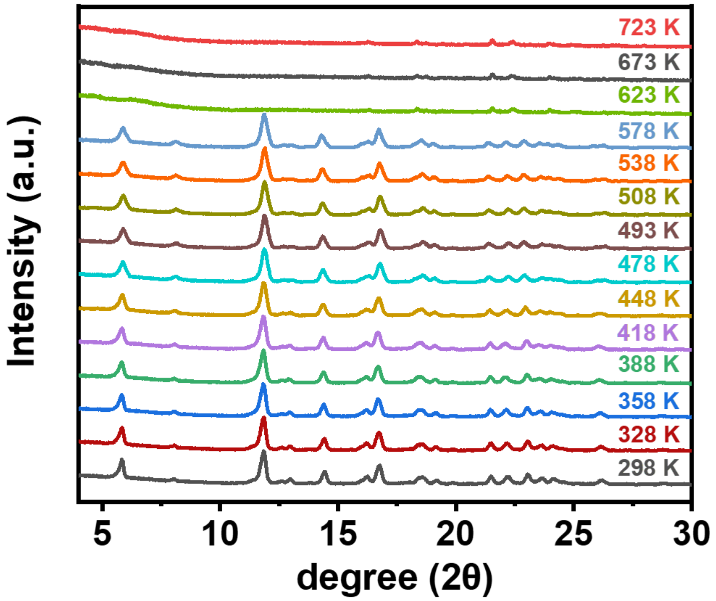

Figure S13. Variable-temperature PXRD analysis of NCU-HOF1a under a N_2 atmosphere.

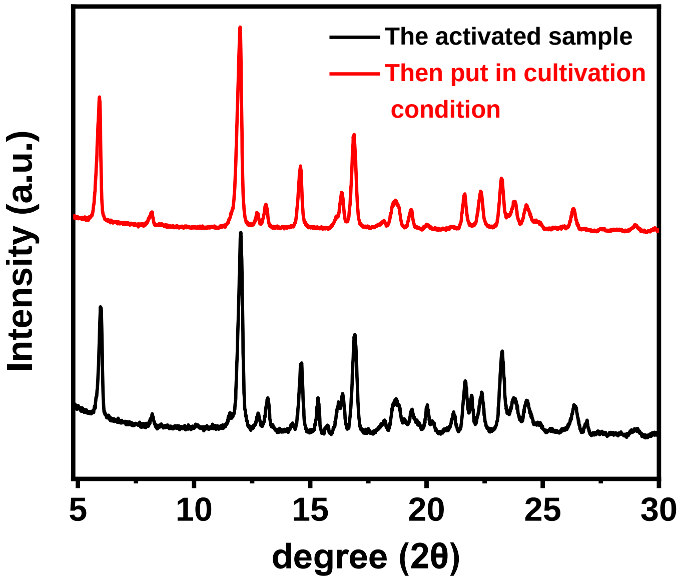

Figure S14. PXRD patterns of NCU-HOF1a before and after immersion under cultivation conditions for 72 hours.

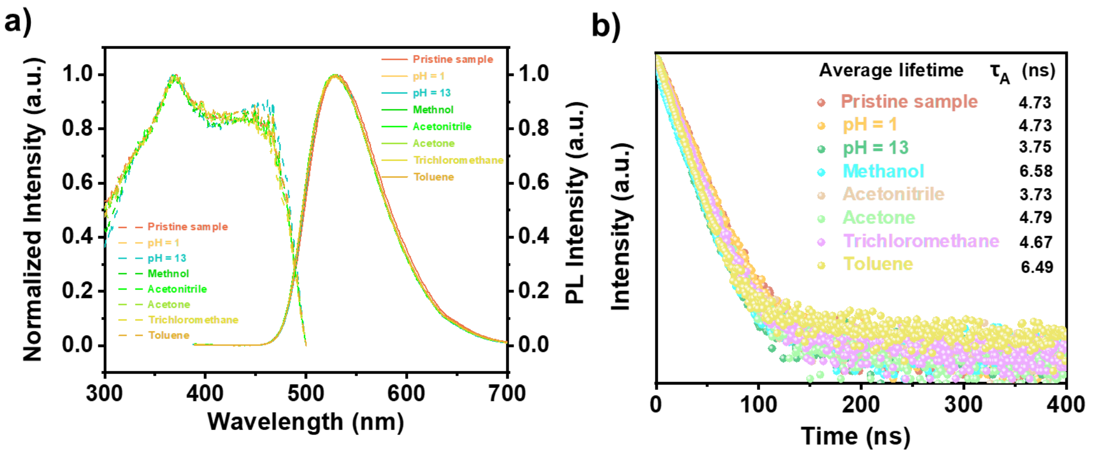

Figure S15. a) Excitation spectra (dotted line) and PL spectra (solid line) of NCU-HOF1a after immersion in different solutions for 72 hours. b) Corresponding fluorescence decay spectra.

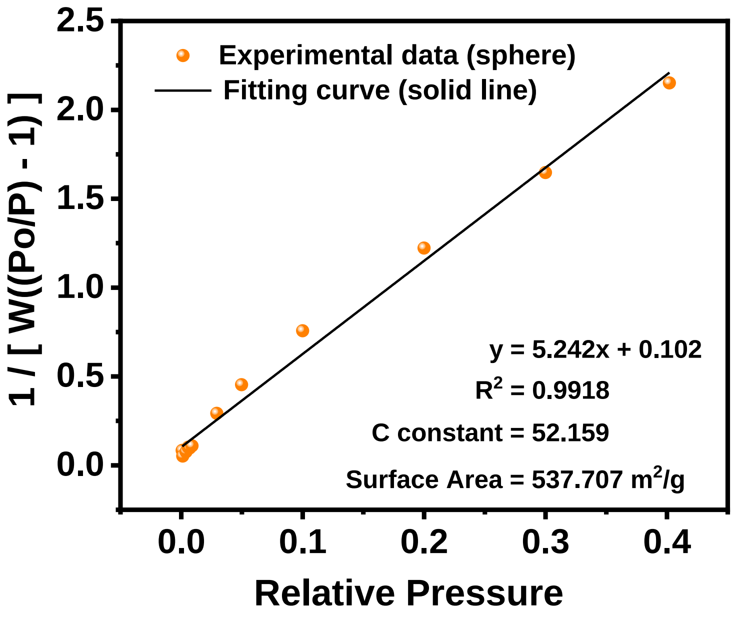

Figure S16. BET fitting curve based on CO_2 adsorption data points.

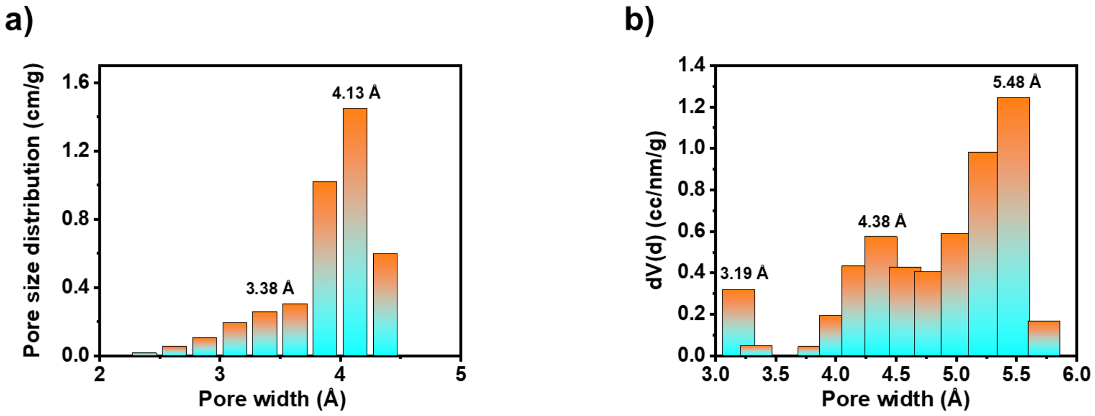

Figure S17. Pore size distribution: (a) calculated using Poreblazer, and (b) measured curve of NCU-HOF1a.

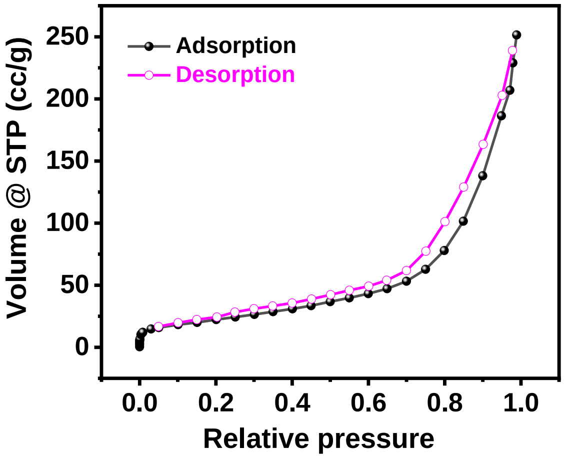

Figure S18. Gas absorption and desorption curves for N_2 in NCU-HOF1a at 77 K.

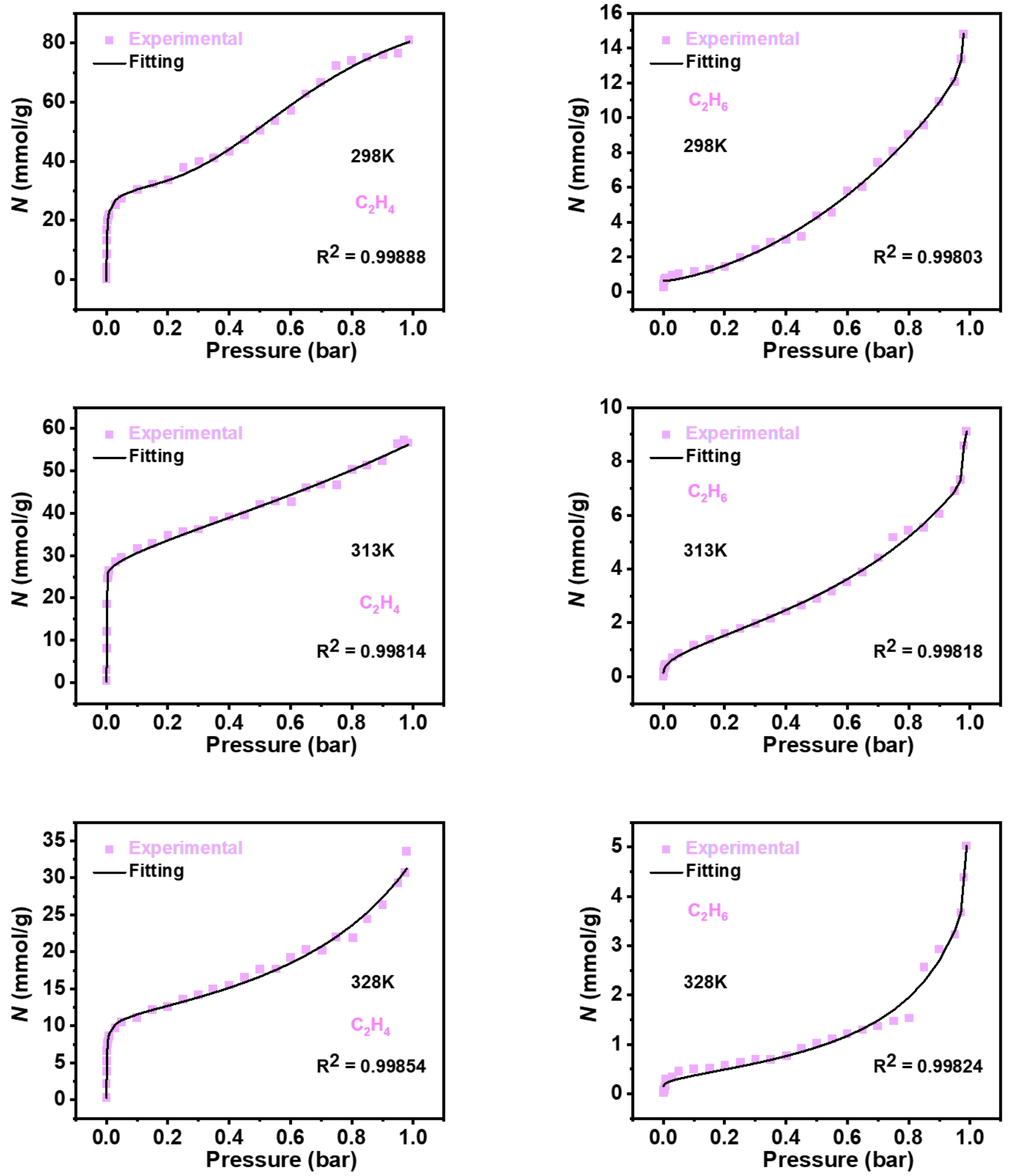

Figure S19. Gas adsorption isotherms for C_2H_4 (left) and C_2H_6 (right) in NCU-HOF1a at a) 298 K, b)318 K and c) 333 K, respectively. Fitting of adsorption curve using the dual-site Langmuir-Freundlich equation (purple dots represent experimental data points, while the black line represents the fitted curve).

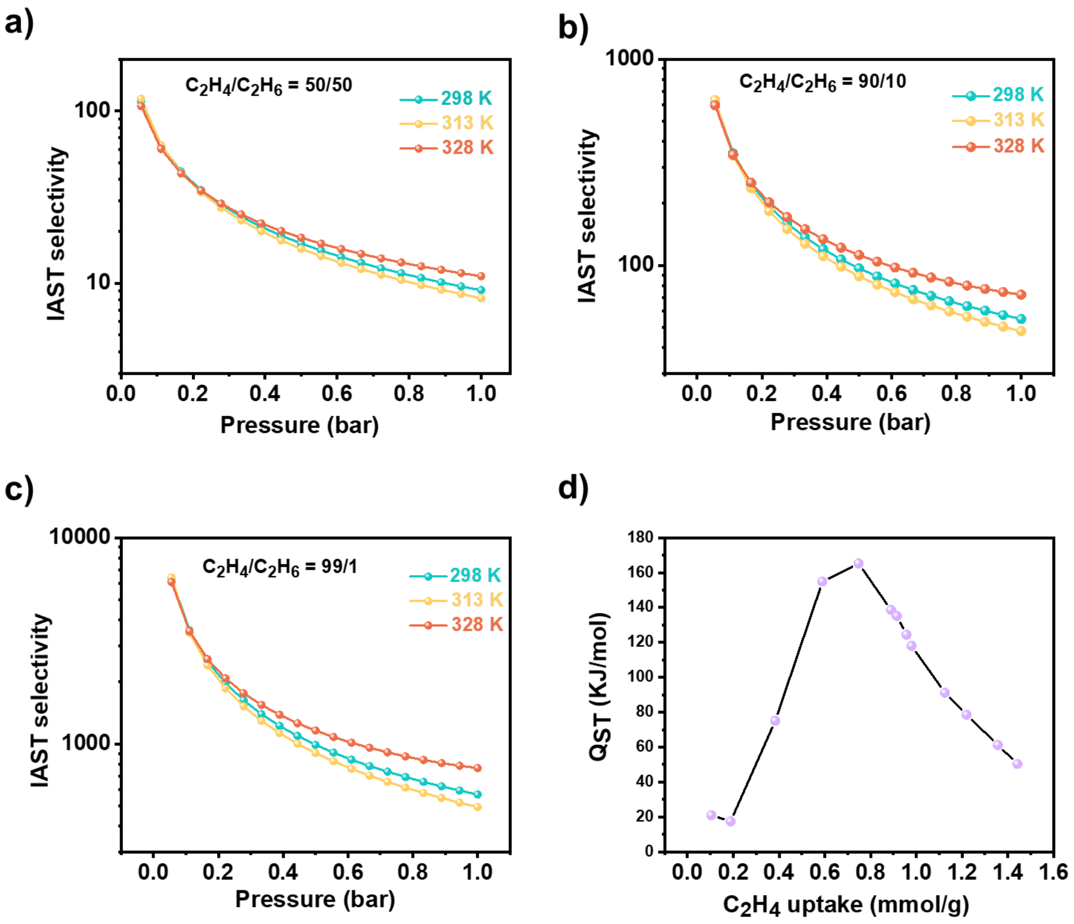

Figure S20. The ideal solution adsorption selectivity under different gas mixture ratios, a) C_2H_4/C_2H_6 = 1:1, b) C_2H_4/C_2H_6 = 9:1, c) C_2H_4/C_2H_6 = 99:1, and different temperatures, and d) Qst curve fitted based on the Clausius-Clapeyron equation.

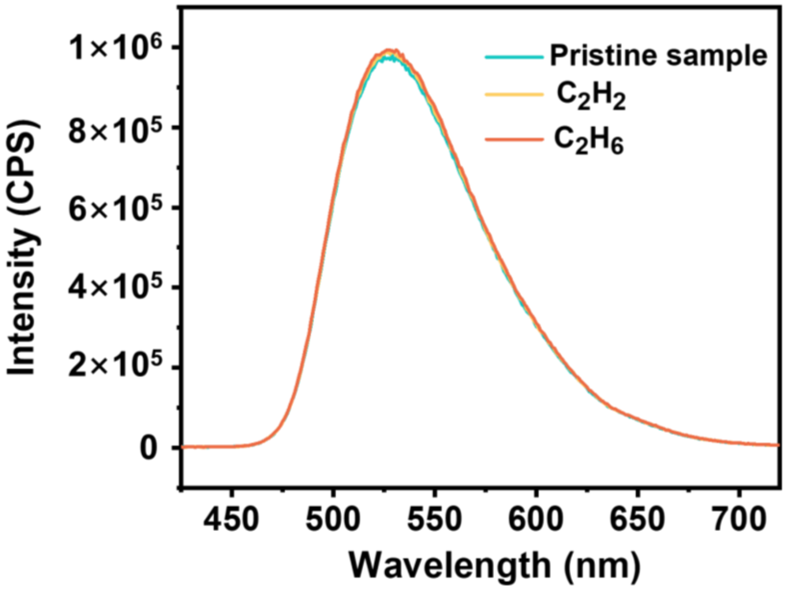

Figure S21. Normalized photoluminescence (PL) spectral changes of NCU-HOF1a equilibrated under C_2H_2 or C_2H_6 pressures (2 bar, 3 hours), λ_{ex} = 368 nm.

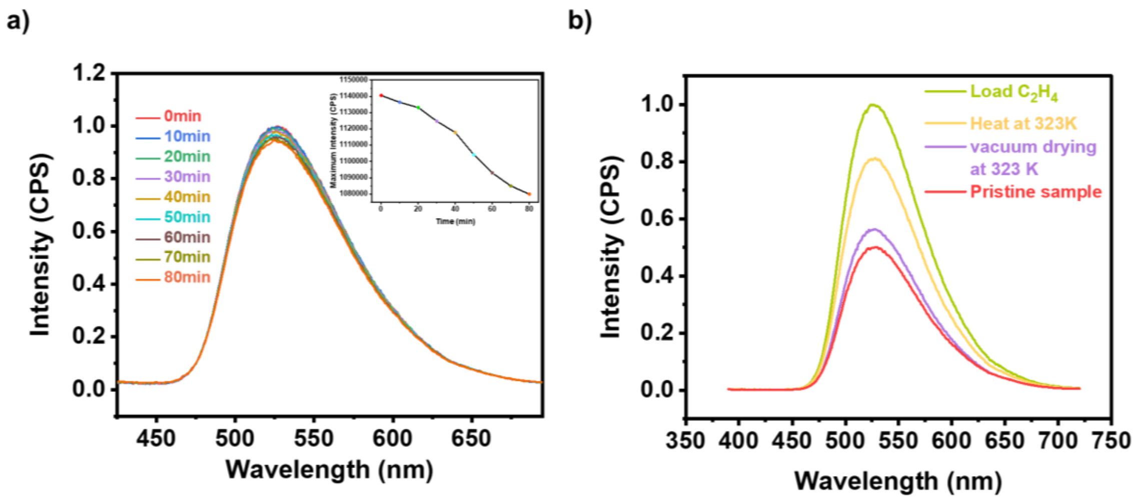

Figure S22. a) Normalized photoluminescence (PL) spectral changes of NCU-HOF1a over time after uptake of C_2H_4 at 298 K, λ_{ex} = 368 nm. Inset: corresponding plots of maximum fluorescence intensity over time. b) Normalized PL spectral changes of NCU-HOF1a (red line), equilibrated under C_2H_4 pressures (2 bar, 3 hours, olive), followed by heating at 323 K (10 min) and vacuum drying at 323 K (10 min, -1 bar).

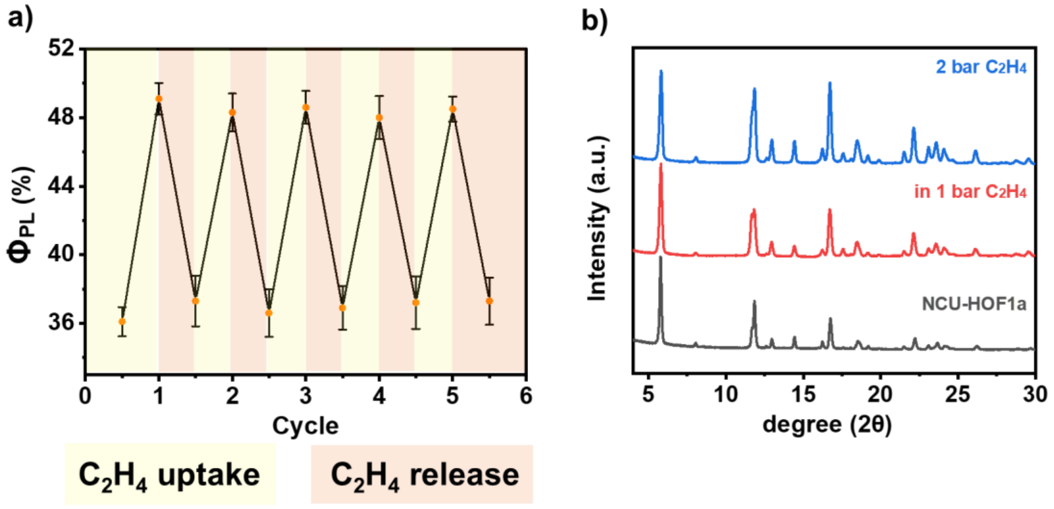

Figure S23. a) Φ_{PL} cycles of NCU-HOF1a with C_2H_4 uptake (equilibrated under C_2H_4 pressures: 2 bar, 3 hours) and release (vacuum drying at 323 K: 10 min, -1 bar). b) PXRD patterns of NCU-HOF1a and those treated under different pressures of C_2H_4 (3 hours).

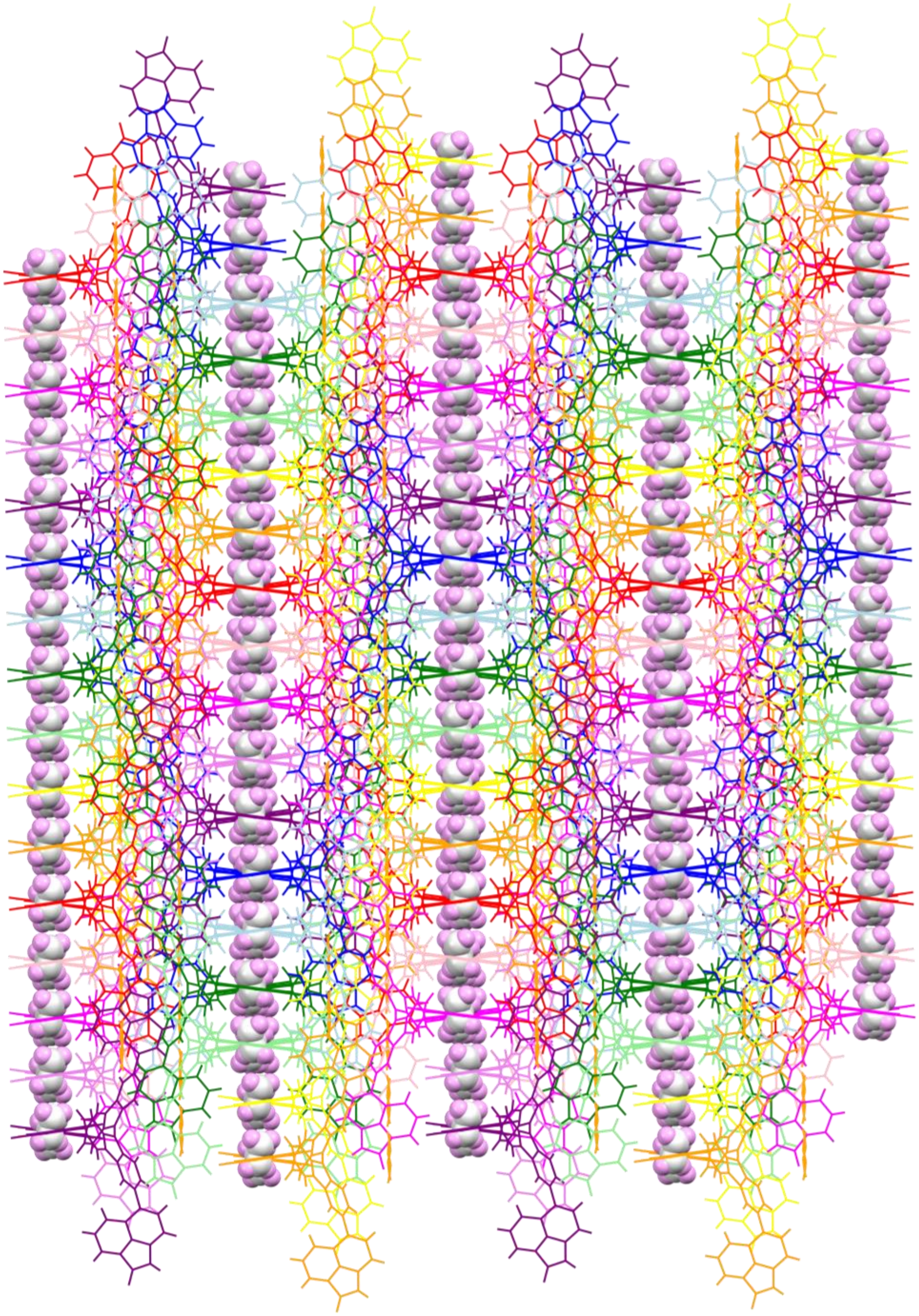

Figure S24. Schematic diagram of structure analysis for NCU-HOF1a@1C_2H_4.

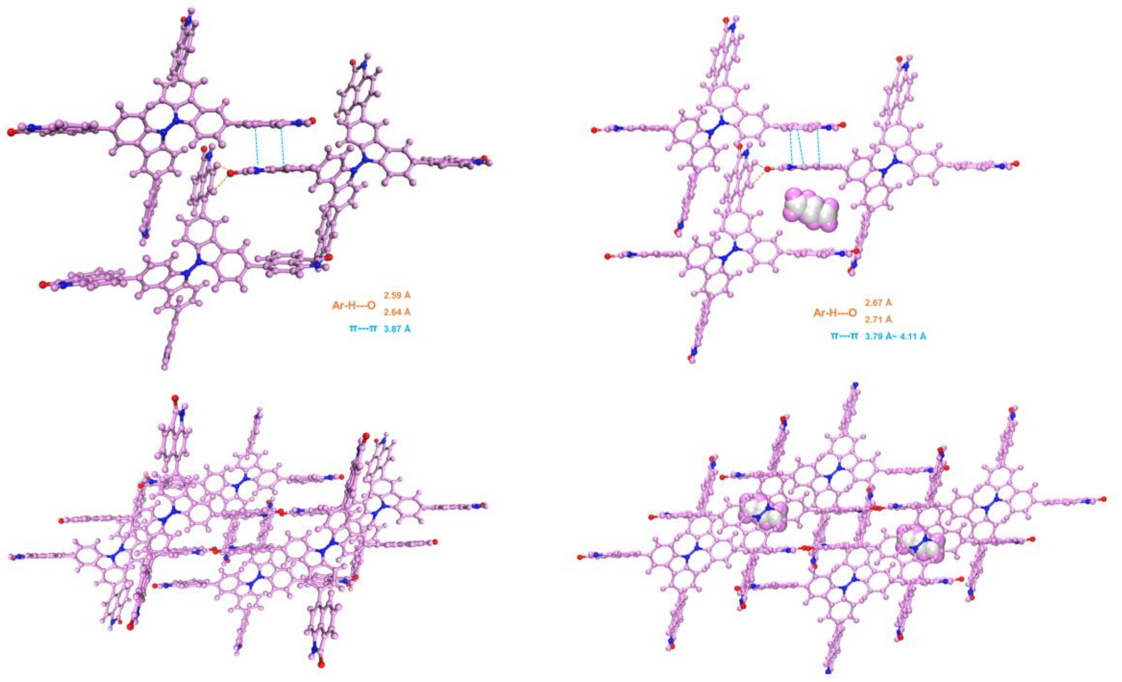

Figure S25. Analysis of intermolecular interactions between adjacent Np synthons for NCU-HOF1a and NCU-HOF1a@1C_2H_4.

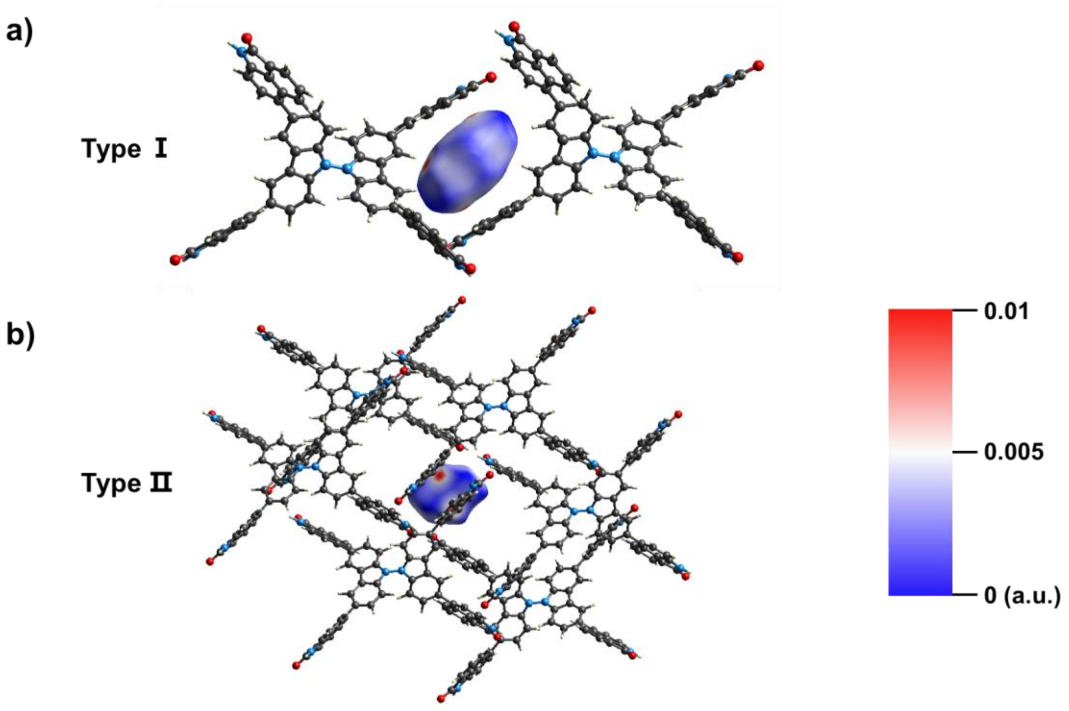

Figure S26. Hirshfeld surface analysis of trapped C_2H_4 gas within NCU-HOF1a@1C_2H_4.

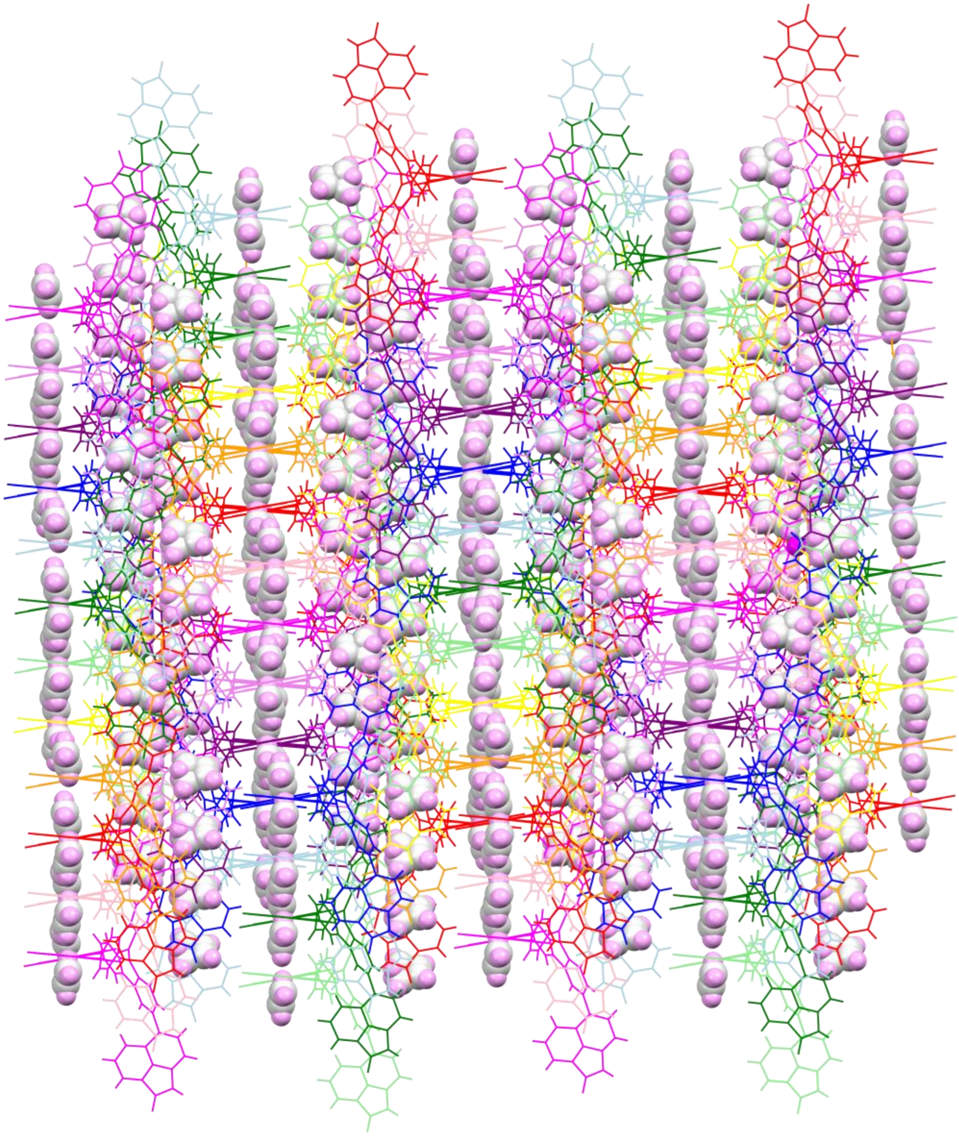

Figure S27. Schematic diagram of structure analysis for NCU-HOF1a@1.75C_2H_4.

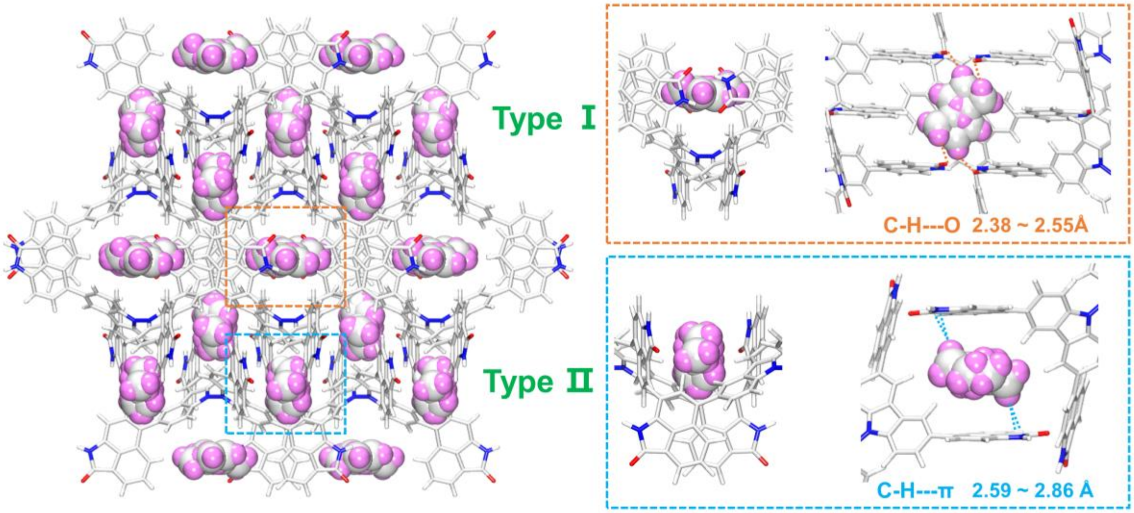

Figure 28. Multiple intermolecular interactions between C_2H_4 gas and Np synthons in the framework with two pore channels of NCU-HOF1a@1.75C_2H_4.

Table S1. Crystallographic data and structural refinement summary.

**Compounds**	**NCU-HOF1**	**NCU-HOF1a**	**NCU-HOF-1a@1C_2H_4**	**NCU-HOF-1a@1.75C_2H_4**
CCDC number	2226443	2226444	2226445	2226446
Empirical formula	$C_{68}H_{36}N_6O_4$	$C_{68}H_{36}N_6O_4$	$C_{68}H_{36}N_6O_4$, C_2H_4	2($C_{68}H_{36}N_6O_4$), 3.5(C_2 H_4)
Formula weight	1001.03	1001.03	1029.08	2100.23
T [K]	193.00	150.00	193.00	275.00
Crystal system	Monoclinic	Monoclinic	Monoclinic	Monoclinic
Space group	I 2/a	C 2/c	C 2/c	C 2/c
a [Å]	16.6654(17)	11.685(3)	11.6021(19)	11.729(3)
b [Å]	38.187(4)	30.037(8)	30.046(4)	30.677(9)
c [Å]	29.129(3)	16.640(4)	16.575(2)	16.645(4)
α [°]	90	90	90	90
β [°]	93.150(7)	109.381(8)	108.819(9)	109.751(10)
γ [°]	90	90	90	90
V [Å^3]	18510(3)	5509(2)	5469.2(14)	5637(3)
Z	12	4	4	2
F (000)	6216	2072	2136	2184
density [g/cm^3]	1.078	1.207	1.250	1.237
μ [mm^{-1}]	0.346	0.388	0.399	0.393
Reflections collected	298664	13692	16530	114357
unique reflections	16920	4506	5721	5844
R (int)	0.1273	0.0512	0.0564	0.0531
GOF	1.163	1.073	1.053	1.013
Radiation	GaK\a, 1.34139	GaK\a, 1.34139	GaK\a, 1.34139	GaK\a, 1.34139
R_1 [$I>2\sigma(I)$] [(a)]	0.1469	0.0636	0.1105	0.0942
ωR_2 [$I>2\sigma(I)$]	0.3193	0.1858	0.2831	0.2587
R_1 (all data)	0.2026	0.0882	0.1740	0.1183
ωR_2 (all data)	0.3485	0.2019	0.3232	0.2843
Largest diff. peak/hole, eÅ^{-3}	0.750/-0.392	0.334/ -0.229	0.531/ -0.200	0.513/ -0.390

Table S2. Representative HOFs with fluorescence behavior.

HOFs	organic ligand	λ_{em}	Φ_{PL}(%)	Ref.
1	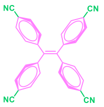	482 nm	\	Nat. Commun., 2022, 13, 1882.
2	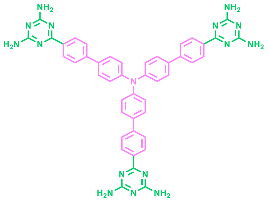	506 nm	\	ACS Appl. Mater. Interfaces, 2021, 13, 32270−32277.
3	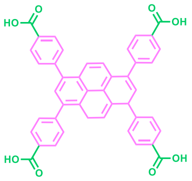	518 nm	\	Angew. Chem. Int. Ed., 2022, 61, e202115956.
4	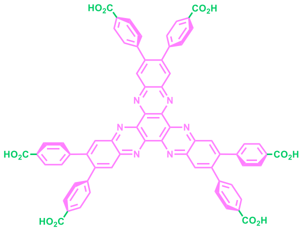	539 nm	\	J. Am. Chem. Soc., 2019, 141, 2111−2121.
5	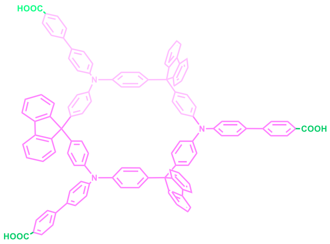	485 nm	21.5	J. Mater. Chem. A, 2023, 11, 4672–4678.
6	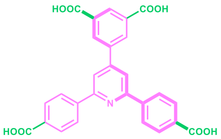	370 nm	\	J. Am. Chem. Soc., 2020, 142, 12478−12485.
7	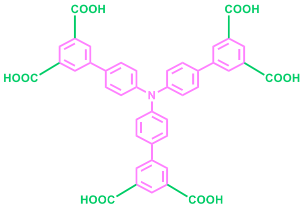	445 nm	21.75	Adv. Optical Mater. 2023, 2202598

Table S3. The specific parameters obtained from the dual-site Langmuir-Freundlich fitting are as follows:

**Temperature**	**Adsorbates**	$\mathbf{q_1^{saturation}}$	$\mathbf{k_1}$	$\mathbf{n_1}$	$\mathbf{q_2^{saturation}}$	$\mathbf{k_2}$	$\mathbf{n_2}$
298 K	C_2H_4	2.76908	12.8772	0.39093	1.67134	1.54261	5.20575
	C_2H_6	29.4128	0.184745	2.35086	0.37034	0.00558182	0.237691
313 K	C_2H_4	60.1652	0.0118995	0.860094	1.16312	1181.57	2.66825
	C_2H_6	20.9295	0.292281	3.67093	1.60339	0.00777078	0.469222
328 K	C_2H_4	99.0391	6.63221e-11	0.203171	28.9046	0.443089	4.68128
	C_2H_6	2.29423	0.732063	8.35498	0.936357	0.0025814	0.455616

Table S4 The photophysical data for equilibrium adsorbed NCU-HOF1 under different C_2H_4 pressures at 298 K.

**C_2H_4 pressure （ bar）**	**λ_{em} [nm]**	**Φ_{PL}[a)] %**	**τ_f [b)] [ns]**		**X^2**	**k_f[c)] [10^7 s^{-1}]**	**k_{nr}[d)] [$10^7 s^{-1}$]**
0	528	36.1	τ_1=3.92 α_1= 95.83 τ_2=8.19 α_2= 4.17	4.28	1.01	8.43	14.93
0.2	527	41.6	τ_1=3.88 α_1= 73.65 τ_2=6.10 α_2= 26.35	4.68	1.00	6.41	14.96
0.4	526	42.7	τ_1=2.57 α_1= -2.2 τ_2=4.95 α_2= 102.2	4.98	1.04	8.57	11.51
0.6	529	42.9	τ_1= 3.49 α_1= 75.79 τ_2= 6.86 α_2= 24.21	4.79	1.08	8.96	11.92
0.8	524	44.2	τ_1=4.02 α_1= 70.31 τ_2=6.24 α_2= 29.69	4.90	1.04	9.02	11.39
1.0	529	45.4	τ_1=4.96 α_1= 87.48 τ_2=7.26 α_2= 12.52	5.36	1.06	8.47	10.19
1.2	525	47.3	τ_1=3.79 α_1= 61.00 τ_2=6.39 α_2= 39.00	5.14	1.05	9.20	10.25
1.4	528	48.7	τ_1=4.70 α_1= 93.99 τ_2=12.56 α_2= 6.01	5.85	1.01	8.32	8.77
1.6	527	48.8	τ_1=5.06 α_1= 94.30 τ_2=13.29 α_2= 5.70	6.19	1.05	7.88	8.27
1.8	527	49.0	τ_1=4.63 α_1= 94.45 τ_2=17.99 α_2= 5.55	7.11	1.04	6.89	7.17
2.0	525	49.1	τ_1=5.05 α_1= 83.34 τ_2=14.51 α_2= 16.66	8.50	1.07	4.94	6.82

[a)] λ_{ex} = 368 nm; [b)] Measured using an integrating sphere method; [c)] Measured using a method of double-exponential decay; [d)] Radiative rate constant ($k_f = \Phi_f / \tau_f$); [e)] Nonradiative rate constant ($k_{nr} = (1-\Phi_f) / \tau_f$).

^{1}H NMR, ^{13}C NMR and MS spectra

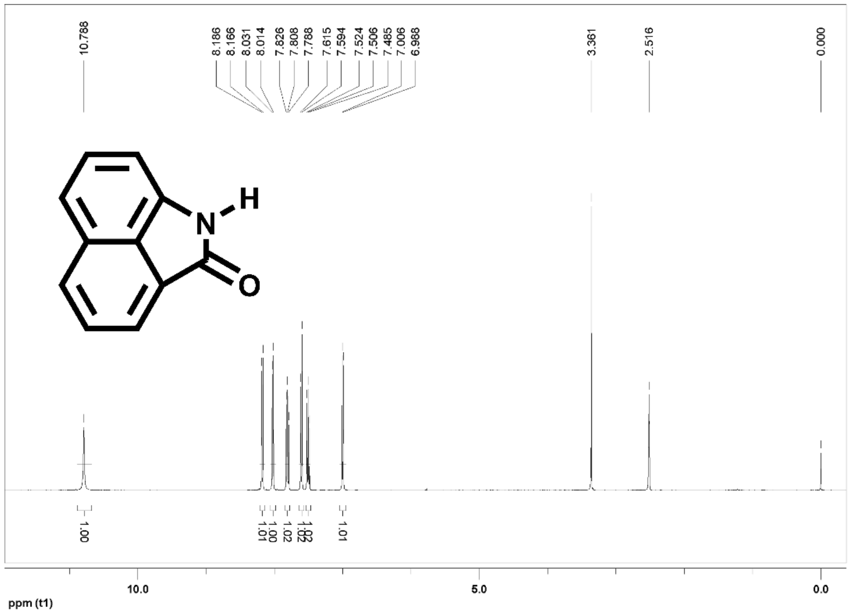

^{1}H NMR spectrum of target **Np** in DMSO-d_6.

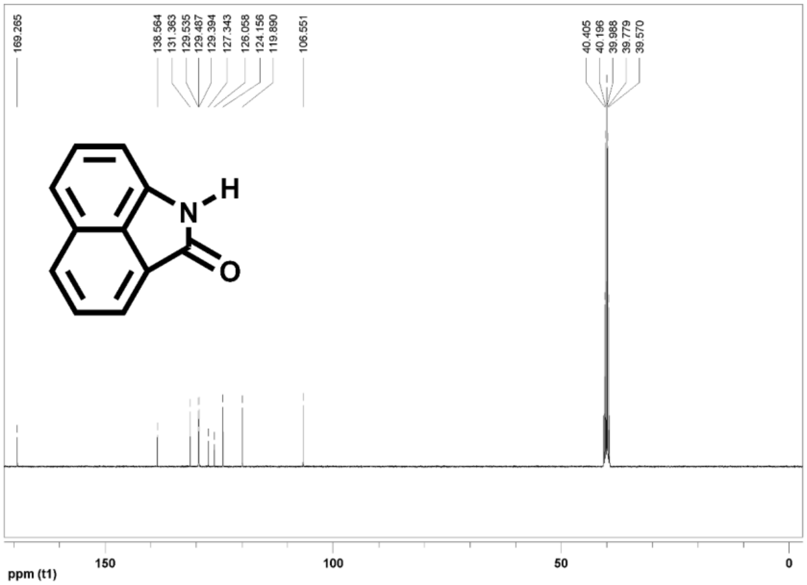

^{13}C NMR spectrum of target **Np** in DMSO-d_6.

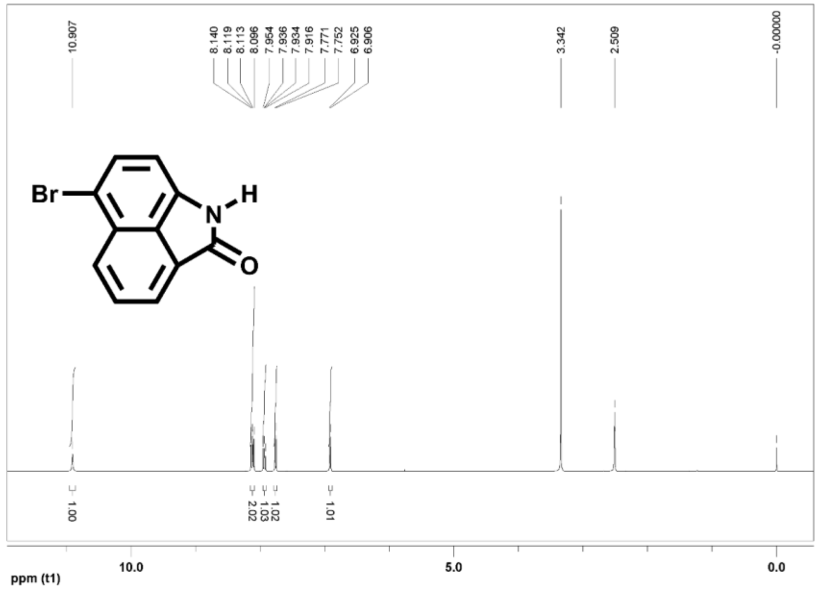

^{1}H NMR spectrum of target **Np-Br** in DMSO-d_6.

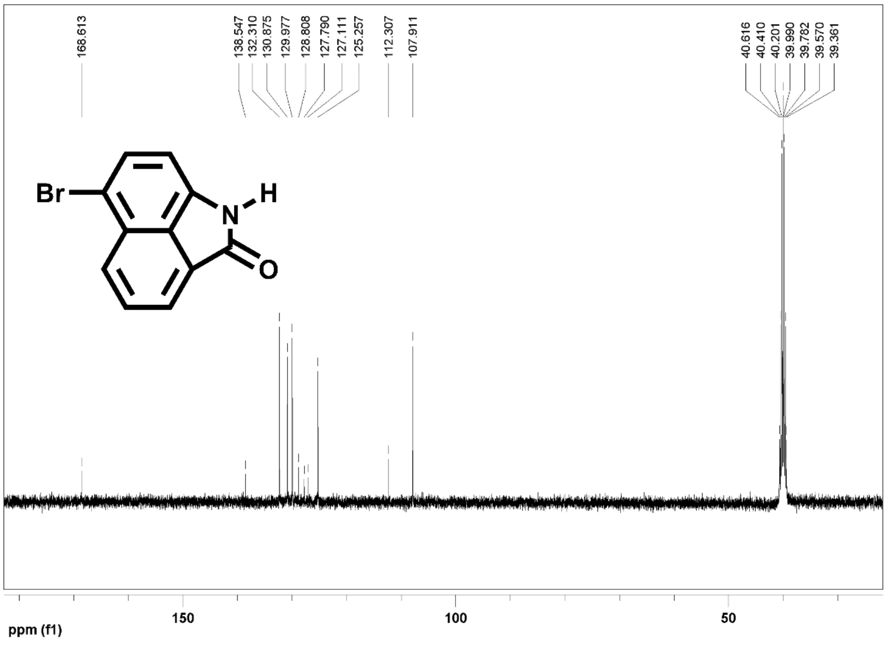

^{13}C NMR spectrum of target **Np-Br** in DMSO-d_6.

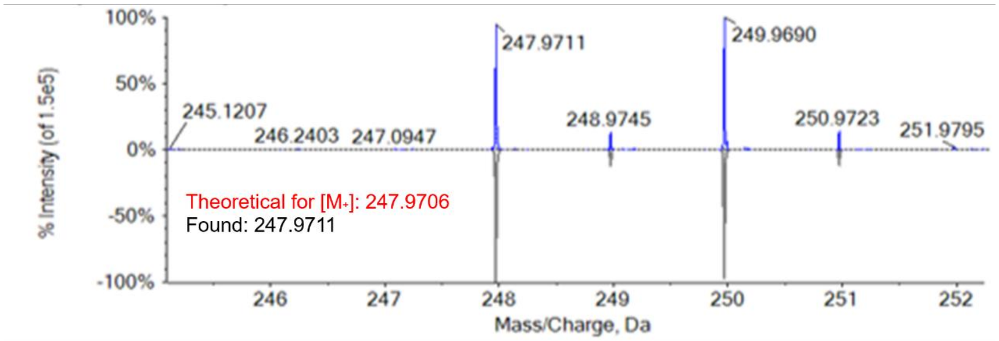

Mass spectrum of target **Np-Br**.

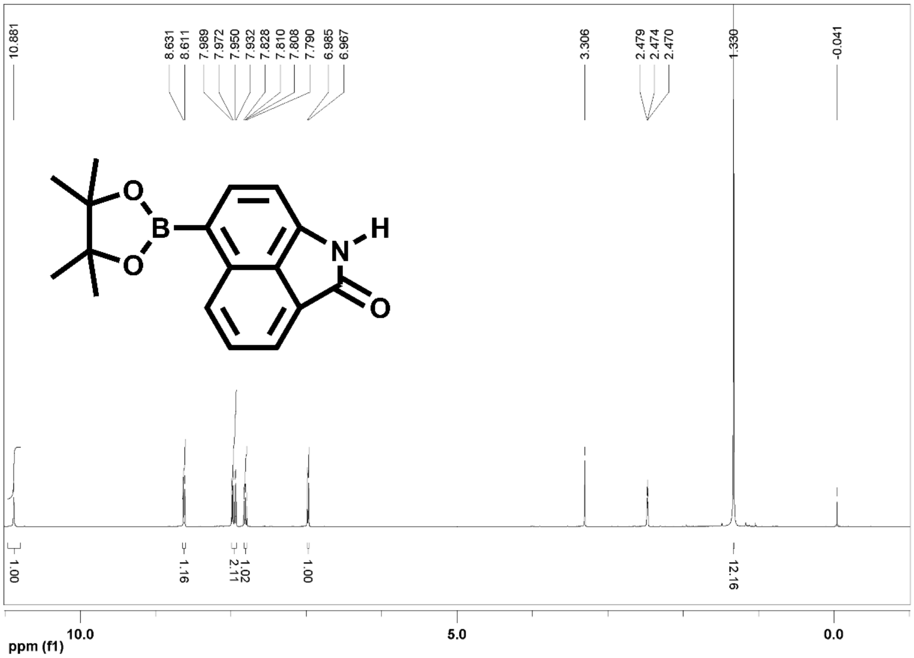

^1H NMR spectrum of target **Np-Pin** in DMSO-d_6.

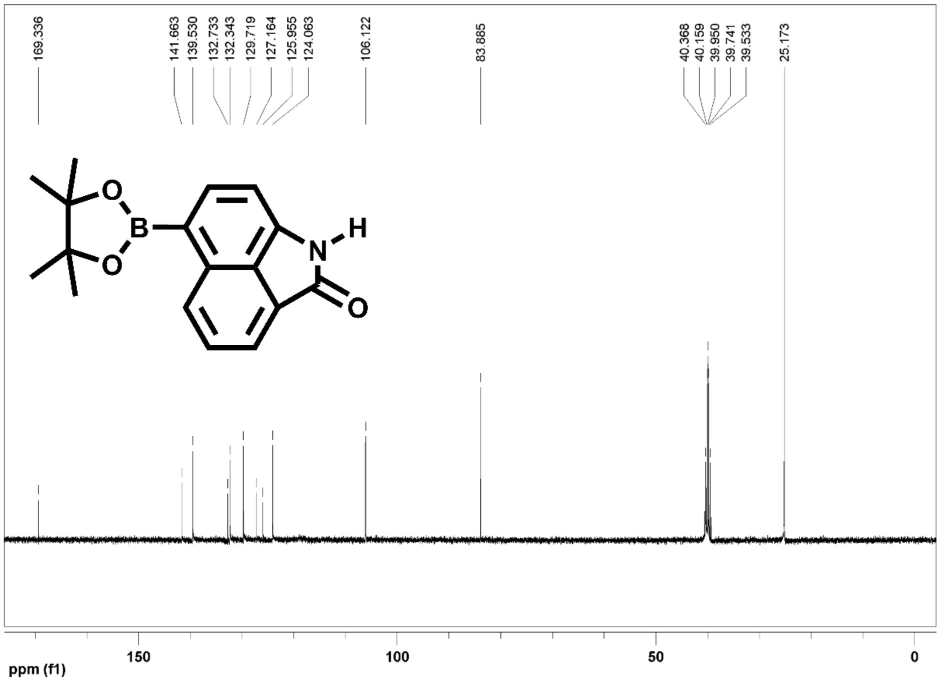

^{13}C NMR spectrum of target **Np-pin** in DMSO-d_6.

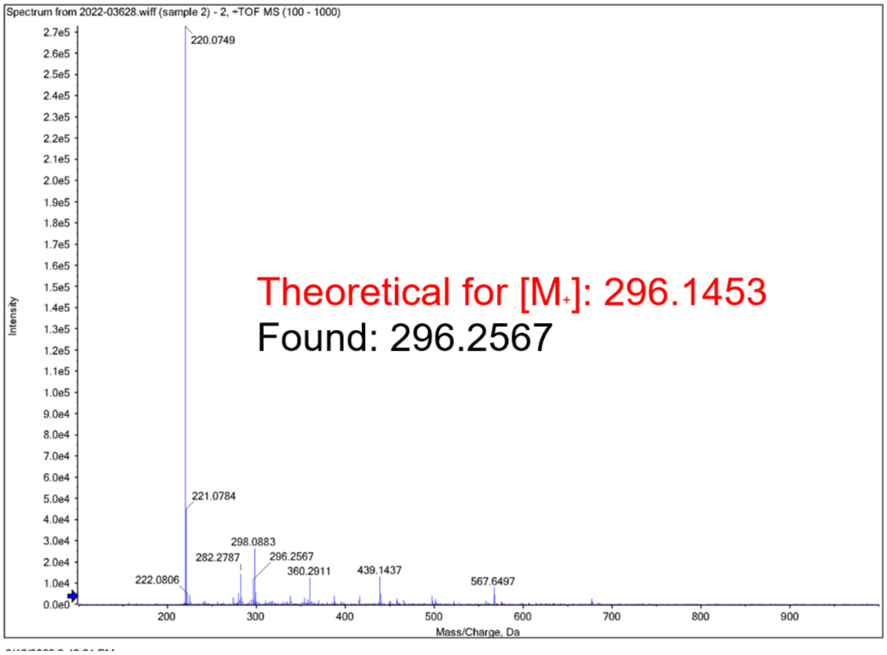

Mass spectrum of target **Np-pin**.

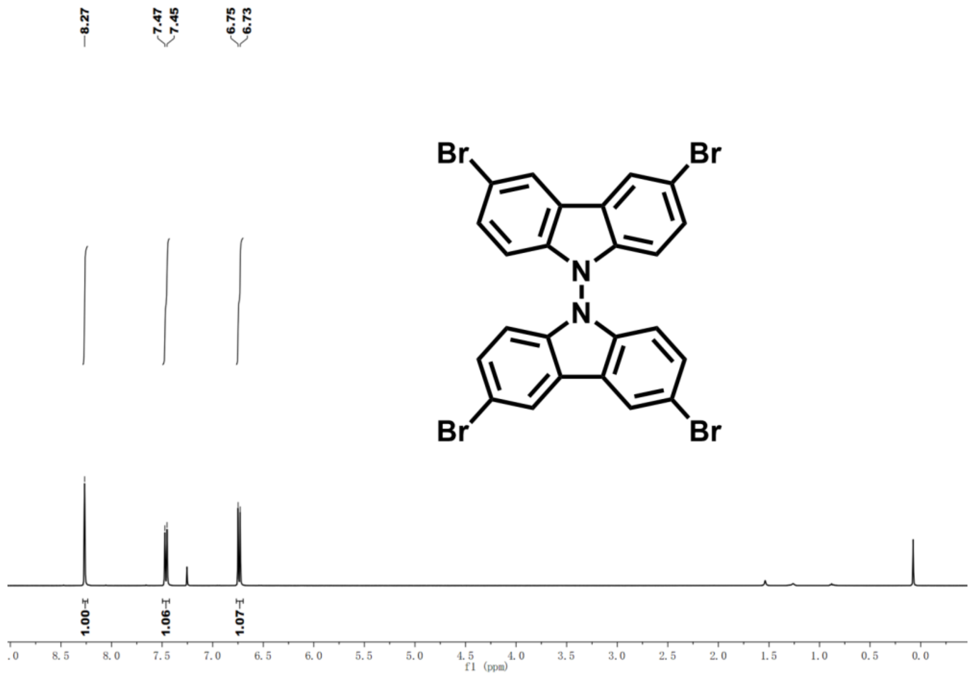

^{1}H NMR spectrum of target **BC-4Br** in $CDCl_3$-d_1.

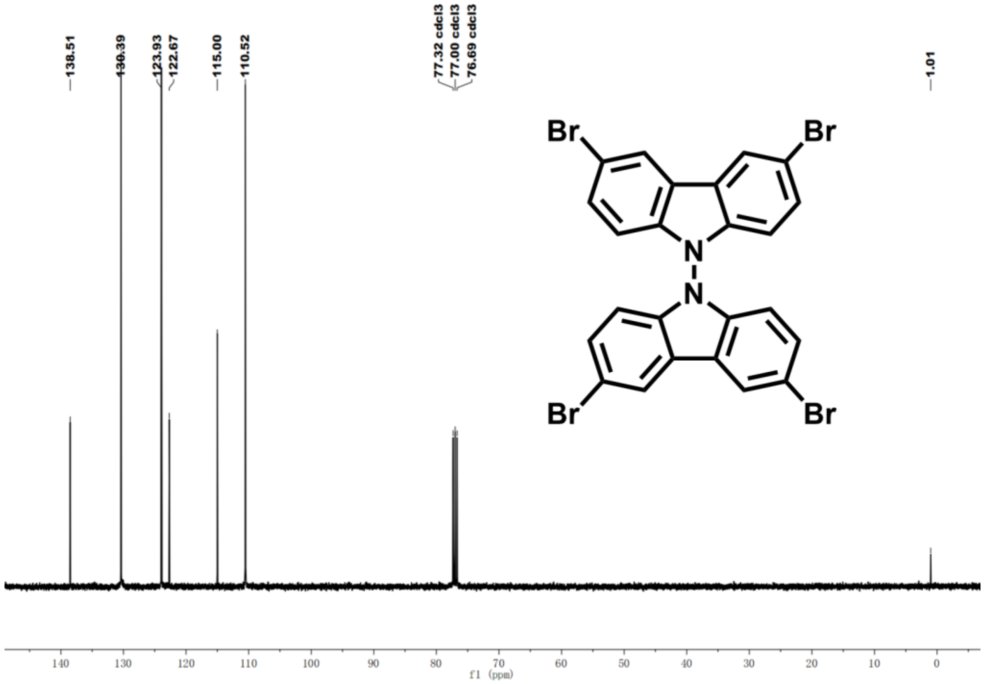

^{13}C NMR spectrum of target **BC-4Br** in $CDCl_3$-d_1.

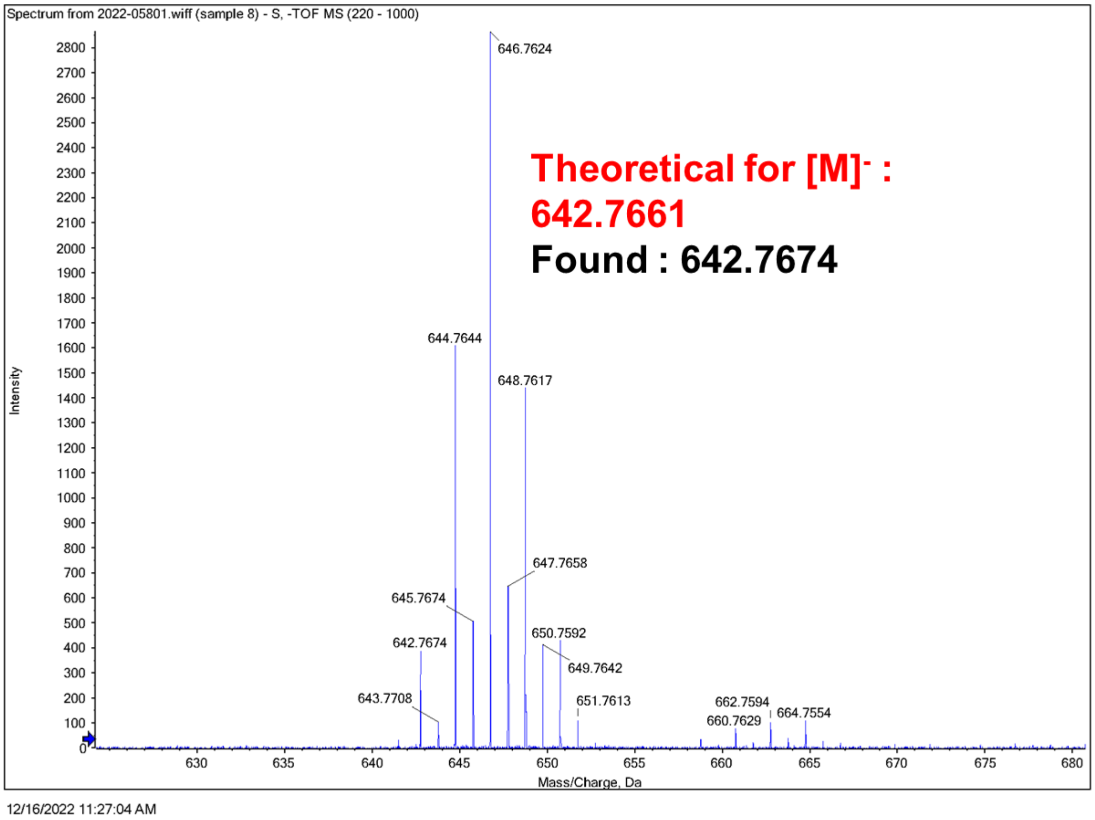

MS spectrum of target **BC-4Br**.

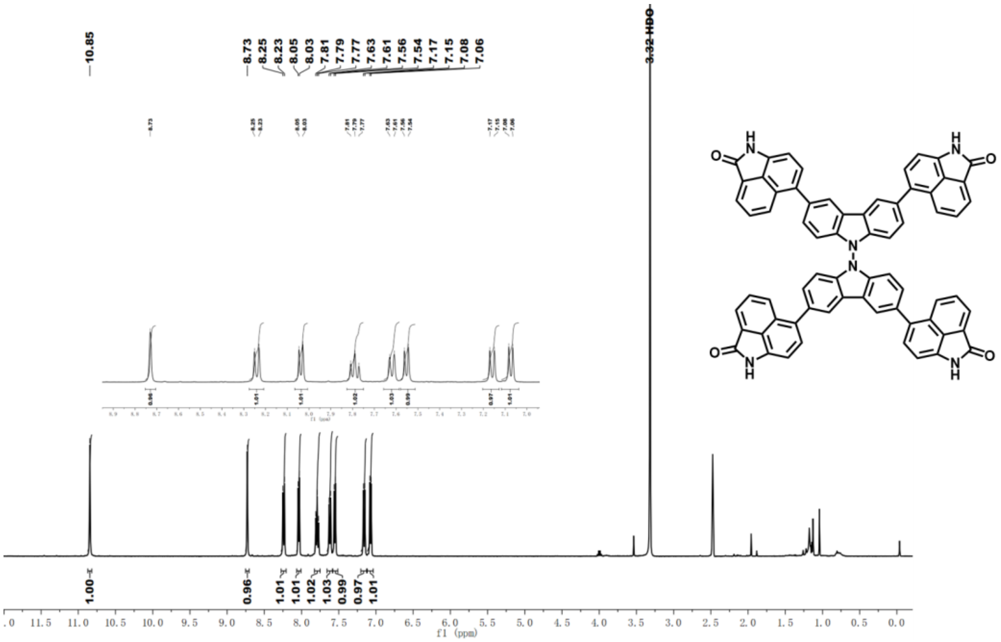

^1H NMR spectrum of target **BC-4Np** in DMSO-d_6.

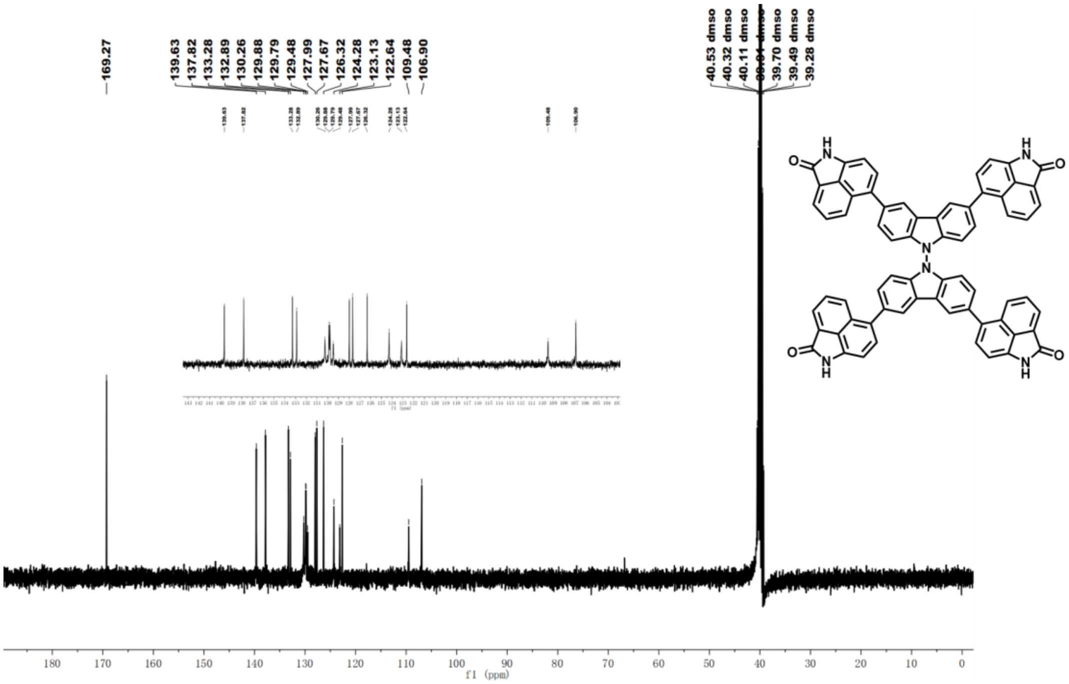

^{13}C NMR spectrum of target **BC-4Np** in DMSO-d_6.

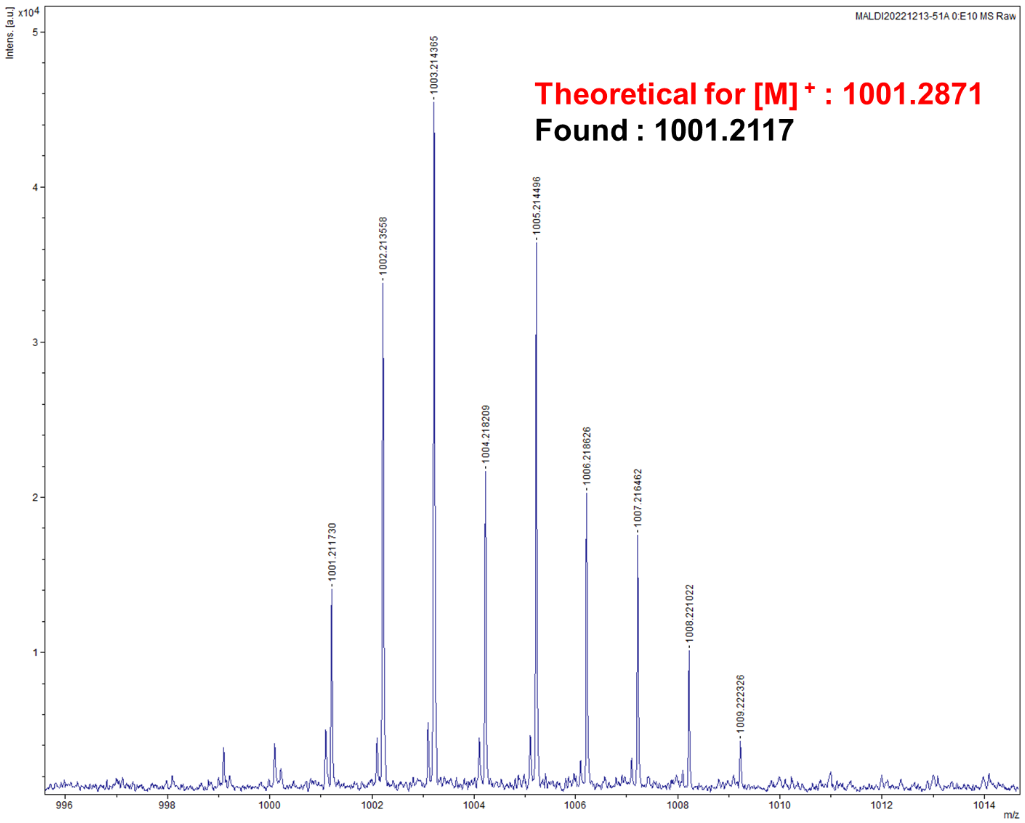

MS spectrum of target **BC-4Np**.

Reference

1. M. J. Frisch, G. W. Trucks, H. B. Schlegel, et al, Gaussian 09 (Revision A.02), Gaussian, Inc., Wallingford CT, 2009.
2. C. T. Lee, W. T. Yang, and R. G. Parr, *Phy. Rev.*, B 1988, **37**, 785.
3. A. D. Becke, *J. Chem. Phy.*, 1993, **98**, 5648-5652.
